# Nitrogen Redox Controls on Greenhouse Gas Production in Yedoma Taliks

**DOI:** 10.1111/gcb.70356

**Published:** 2025-07-21

**Authors:** Oded Bergman, Katey Walter Anthony, E. Eliani‐Russak, Orit Sivan

**Affiliations:** ^1^ Department of Earth and Environmental Sciences Ben Gurion University of the Negev Beersheva Israel; ^2^ Water and Environmental Research Center University Alaska Fairbanks Fairbanks Alaska USA

**Keywords:** biogeochemistry, methane, *Methanoperedens*, N‐AOM, nitrous‐oxide, permafrost, soil microbiology, Thermokarst, Yedoma

## Abstract

Large carbon and nitrogen pools are disproportionately concentrated in the icy, Pleistocene‐aged silt deposits of Arctic Yedoma permafrost. Upon thaw, these undergo microbial mineralization, releasing greenhouse gases (GHGs) including carbon dioxide (CO_2_), methane (CH_4_), and nitrous oxide (N_2_O). Here, we present combined geochemical data with microbial function and community dynamics from deep (7‐m) talik soil boreholes in water‐unsaturated yedoma upland in interior Alaska. Our results reveal significant in situ seasonal shifts in microbial function, community composition, and diversity throughout the talik. Methanogenesis persisted in the deep talik year‐round. Winter methanotrophy was negligible within and above the methanogenic zone, leading to elevated CH_4_ production and emission to the atmosphere. This is likely due to reduced microbial methanotrophic activity associated with lower temperatures and nitrogen availability. During summer, strong aerobic methanotrophy near the soil surface reduced CH_4_ emissions. Nitrate/nitrite‐mediated anaerobic oxidation of methane (AOM) by both archaea (ANME‐2d clade) and bacteria (NC10 phylum) occurred at and above the anoxic methanogenic zone, further offsetting CH_4_ production. In contrast to CH4 production potentials, which were higher in surface soils in winter compared to summer, we observed higher N_2_O production potentials in summer compared to winter. Nitrous oxide concentrations peaked at 10 cm (7.2 μM) and 105 cm (6.7 μM) and were associated with denitrification; nitrogen‐mediated AOM by *Methanoperedens* (ANME2d). In the summer only and within the top 1 m of soil, high expression of nitrogen‐related genes (narG, norB, amoA, Annamox, and Feammox) indicated active redox dynamics, potentially providing nitrogen species for AOM. The potential N_2_O production in summer may imply higher net GHG emissions from yedoma uplands as climate change leads to longer summers and warmer soils in the future.

## Graphical abstract

Arctic Yedoma permafrost holds large carbon and nitrogen pools that, when thawed, undergo microbially mediated processes that release greenhouse gases like methane and nitrous oxide. We analyzed seasonal shifts in microbial community dynamics and geochemistry in deep thawed soils (talik) and found year‐round methane production, with limited winter methane consumption and corresponding elevated methane emissions to the atmosphere. In summer, increased methane oxidation and active nitrogen cycling coincided with higher nitrous oxide production. These findings suggest that longer, warmer summers may shift greenhouse gas emissions from predominantly winter methane to increased summer nitrous oxide release in Yedoma uplands.

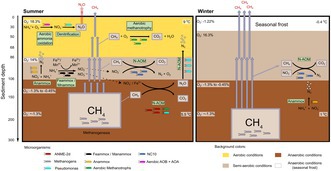

## 1 Introduction

Permafrost soils cover about a quarter of the northern hemisphere land surface. Approximately, 25% of the permafrost soil carbon pool is concentrated in silty, ice‐rich, Pleistocene‐aged yedoma permafrost in Siberia and Alaska, which makes up 4%–7% of the permafrost region (Hugelius et al. 2014; Strauss et al. 2017, 2021). The carbon pool of terrestrial permafrost soils is estimated between 1330 and 1580 Pg (Schuur et al. 2015), including 327–466 Pg in yedoma (Strauss et al. 2017). While less definitive, the nitrogen pool is estimated at 41.2 Pg at the upper 20 m of the yedoma domain (Strauss et al. 2022).

Climate warming leads to permafrost thaw and the formation of intra‐permafrost zones of perennially thawed soil, termed taliks (Farquharson et al. 2022; Parazoo et al. 2018; Romanovsky et al. 2010; Turetsky et al. 2020). Upon thaw, permafrost soil carbon and nitrogen reservoirs may become available for microbial mineralization (Burnett et al. 2022; Ramm et al. 2022; Wegner et al. 2022), leading to the production and emission of atmospheric CO_2_, CH_4_, and N_2_O in yedoma landscapes as well as other non‐yedoma tundra, peatlands, ponds, and lakes (Abnizova et al. 2012; Elberling et al. 2010; Strauss et al. 2022; Voigt et al. 2017, 2020; Walter Anthony et al. 2016; Wang et al. 2023; Yang, Peng, Marushchak, et al. 2018) in the arctic and boreal regions. Methane and N_2_O are potent ozone‐depleting greenhouse gases (GHGs) with a global warming potential roughly 33 and 300 times that of CO_2_ over a 100‐year timescale (Shindell et al. 2014).

Microbial communities differ significantly in composition and structure across various permafrost environments, presenting dominant microbial groups, such as *Proteobacteria* and *Actinobacteria* (Hultman et al. 2015; Jansson and Taş 2014; MacKelprang et al. 2011, 2017; Steven et al. 2008; Yergeau et al. 2010). Concomitantly, community shifts have been related to permafrost thaw in vivo and in incubation experiments (Barbato et al. 2022; Chen et al. 2021; Ji et al. 2020; MacKelprang et al. 2011; Monteux et al. 2018; Romanowicz et al. 2023). The widespread distribution of methanogenic and methanotrophic microbial communities in permafrost is also well documented, together with shifts in composition related to permafrost thaw and talik formation (Ganzert et al. 2007; Holm et al. 2020; MacKelprang et al. 2011; Mondav et al. 2014; Singleton et al. 2018; Waldrop et al. 2023; Walter Anthony et al. 2024). Methanotrophy in permafrost sediments is performed by both anaerobic archaea (ANMEs) and aerobic bacteria (mainly Gammaproteobacteria) (He et al. 2022; Lotem et al. 2023; Martinez‐Cruz et al. 2018; Winkel et al. 2018, 2019).

Anaerobic oxidation of CH_4_ (AOM) can be coupled to N‐AOM. For example, AOM coupled to nitrate (NO_3_^−^) and nitrite (NO_2_^−^) reduction has been shown by anaerobic bacteria including the NC10 phylum, in both freshwater and marine sediments (Ettwig et al. 2010; He et al. 2022; Kits et al. 2015; Li et al. 2024; Lomakina et al. 2020; Oswald et al. 2017). Under such settings, methanotrophy can co‐occur alongside N_2_O production (Hao et al. 2022). While N_2_O emissions were measured from thermokarst mounds in North‐Siberia, a region of continuous permafrost where upland taliks have yet to develop (Marushchak et al. 2021), to our knowledge, the potential for N_2_O production in warmer regions, such as interior Alaska, has never been explored. While N and C mobilization are interconnected through microbial and ecosystem processes, their dynamics are complex and require thorough investigation (Monteux et al. 2018; Strauss et al. 2024).

This study focuses on the seasonal couplings between the CH_4_ and nitrogen cycles, and potential N_2_O production, in unsaturated yedoma upland taliks in interior Alaska. Our warm, interior‐Alaska study site, with advanced permafrost thaw, allows exploration of the potential carbon and nitrogen dynamics in the yedoma domain, as climate warms and permafrost degradation leads to talik development in colder Arctic regions in the future (Walter Anthony et al. 2024). The primary goal of this study was to capture the seasonal variation in microbial function and community composition in upland yedoma soils. A secondary aim focuses on spatial variations within our study site. Towards the first goal, we analyzed a 7‐m deep soil profile of the full talik down to the top of permafrost. For the secondary goal, we compared soil cores from mid and high elevations at the study site, whereby elevation corresponded to the depth of the surface aerobic zone.

**FIGURE 1:**
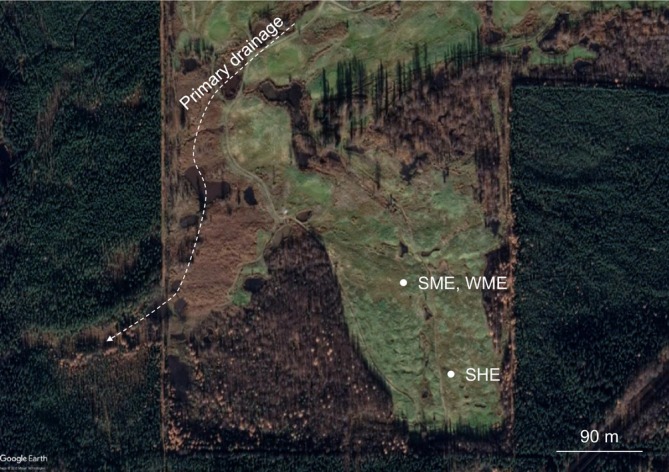
Map of the study site. Located at North Star Yedoma (NSY), 7 km northwest of Fairbanks, Alaska. Three boreholes were drilled and sampled. During summer (September 15, 2021) two shallow boreholes were excavated: SME = Summer Mid Elevation (3.2 m), SHE = Summer High Elevation (2.3 m). One deep borehole was drilled during winter (March 18, 2023): WME = Winter Mid Elevation (7.25 m).

Our study site, informally named North Star Yedoma (NSY) (Figure 1), is characterized by conical thermokarst mounds, soil‐mound features that form by melting of polygonal ground‐ice wedges in yedoma (Murton et al. 2015; Péwé 1954). Geophysical surveys indicate a 5–9 m thick talik across the NSY study field (Walter Anthony et al. 2024). To achieve our goals, we analyzed three soil boreholes collected from NSY (Figure 1), utilizing a combination of soil geochemical methods, together with advanced molecular techniques.

## 2 Materials and Methods

### 2.1 Study Site

North Star Yedoma (NSY) is located in interior Alaska (Latitude: 64.8939, Longitude: −147.6373), seven kilometers northwest of Fairbanks in a region of discontinuous permafrost. Originally a mature black spruce forest, this large open field contains thermokarst features, formed following the anthropogenic removal of the forest and surface organic soil horizon in 1978. In the 1990s, the eastern half of the field was seeded with turf grasses for the establishment of a rugged golf course. The western side was unseeded, allowing natural forest succession. Over the last 20 years, ground‐ice melt and thermokarst subsidence continued across the whole field, resulting in the mound‐ridden surface at NSY today. Regional borehole data indicate icy, organic‐rich yedoma silts extending over 40 m below ground. The NSY study site is extensively described in Walter Anthony et al. (2024), including vegetation, soil description, disturbance history, etc.

### 2.2 Sample Collection, Preparation, and Physicochemical Analysis

We collected soil core samples during late summer (September 15, 2021) by drilling boreholes on the tops of two thermokarst mounds in different elevational locations (Figure 1). The first was located at mid elevation (SME = Summer Mid Elevation, id BH1, 3.2 m depth) and the second near the highest elevation in the field (SHE = Summer High Elevation, id BH2, 2.3 m). Boreholes were drilled using a gas‐powered AMS frozen soil auger with 5 cm of internal (core) diameter. On March 18, 2023, a winter core (WME = Winter Mid Elevation, id BH6, 7.25 m) was drilled 60 cm away from BH1, using a Talon Drill system. SHE is at about 623 ft elevation, SME is about 609 ft elevation, and the drainage path at the valley bottom is 585 ft. Subsamples from the summer core, not volumetrically controlled, were collected at 10–15‐cm intervals, placed in ziploc bags, and transferred to the freezer. Winter core sampling was volumetrically controlled, allowing for determination of soil dry density and degree of water saturation (see Walter Anthony et al. 2024 for details).

### 2.3 Environmental Parameters

The following physicochemical parameters were measured: Volumetric water content (VWC) gravimetric water content (GWC), CH_4_ and CO_2_ concentrations, organic carbon (C_org_, %), organic nitrogen (N_org_, %), δ^15^N (‰), δ^13^C (‰). The full, detailed description of the methods used is presented in Walter Anthony et al. (2024). Briefly, GWC was determined as the weight loss after drying at 105°C, expressed as a percentage of wet weight. VWC was calculated as the product of gravimetric moisture content and dry density. Where dry density was not measured, site‐specific or depth‐dependent relationships were used.

To measure dissolved gases (CH_4_ and CO_2_), 3 mL soil plugs were collected from each depth using modified syringes and transferred, in the field, into 20 mL glass serum vials pre‐filled with sterile 5 M NaCl solution. Vials were immediately sealed airtight without headspace using butyl rubber stoppers and aluminum crimps, and stored inverted until processing. Gas concentrations (CO_2_, CH_4_, and N_2_O) were analyzed using a focus gas chromatograph (GC) system (Thermoscientific, Germany) equipped with a flame ionization detector and shinCarbon ST packed column (Restek, USA). C_org_ and N_org_ were measured using an elemental analyzer (Costech ECS4010) coupled to a Finnigan DeltaPlus XP Isotope ratio mass spectrometer (Thermo Scientific). Samples were acidified with 31.45% HCl to remove inorganic carbon, rinsed, and dried prior to analysis. C_org_ and N_org_ concentrations were reported in weight percentage (wt%). δ^13^C and δ^15^N isotopic compositions were measured simultaneously during the analysis and are expressed in per mil (‰) relative to V‐PDB and ambient air, respectively. For the full methodological details, see Walter Anthony et al. (2024).

Near our mid‐elevation borehole site, NSY soil temperatures were measured with thermistors at four depths (10, 100, 150, 200, and 250 cm) below the ground surface at one‐hour intervals by a HOBO datalogger (4‐channel UX120‐006) wired to TMCx‐HD thermistor temperature sensors (Onset Computer Corp; accuracy 0.10°C). Oxygen concentrations (as %) were measured using Apogee SO‐411 sensors. Two sensors (at 10, 50) were installed in the thermokarst‐mound flank, and two additional sensors were installed next to a mound top at 100 cm and 190 cm depth on May 23, 2023. As temperature and oxygen were only measured at discrete depths and not throughout the depth profile, they were not used in the PCA analysis (see below).

### 2.4 PCA Analysis

We sought to characterize the differences in various environmental conditions related to sampling season (SME vs. WME samples, *n* = 15, up to ~3 m), depth of the full talik profile (WME—shallow (< 3 m) versus deep (> 3 m) samples, *n* = 11, up to ~7 m) of and elevation (SME vs. SHE, *n* = 16, up to ~3 m). We performed for all three separate PCR analyses on all samples. We included in the analyses eight environmental parameters: GWC, CH_4_, and CO_2_ concentrations, C_org_, N_org_, C_org_/N_org_ ration, δ^15^N, and δ^13^C. Prior to analysis, all samples were centered and scaled (log10 + 1 values). Correlation‐metrices and loading scores are presented in Tables S2–S4. As oxygen and temperature sensors were located at discrete depths, different from the sampling depth, they were excluded from the PCA analysis.

### 2.5 DNA Extraction

Soil samples from BH1 (23, 56, 68, 86, 106, 166, 186, 259, 295, and 305 cm), BH2 (8, 38, 84, 146, 195 and 225 cm) and BH6 (50, 100, 162, 198, 285, 345, 535, 564, 655, 695 and 711 cm) were taken for molecular microbial analysis. DNA was extracted from approximately 0.25 g soil, using the PowerSoil DNA Isolation Kit (QIAGEN, Hilden, Germany; formerly MoBio, CA, USA), according to the manufacturer's instructions, with the following modifications: After lysis buffer addition, samples were incubated at 65°C for 10 min to enhance lysis. The elution buffer was pre‐warmed at 70°C for 5 min to improve DNA recovery. DNA extracts were subsequently stored at −80°C until use.

### 2.6 Real Time PCR (qPCR)

qPCR was performed using a CFX Duet qPCR instrument (Bio‐Rad, USA) and analyzed with the CFX Maestro software. Reactions targeted genes associated with the following processes: methanogenesis (mcrA), methane oxidation (pmoA), anammox (hzsB), Feammox (16S gene for *Acidimicrobiaceae* bacterium A6), aerobic ammonium oxidation (amoA, bacterial and archaeal), NC10 phylum (16S gene), and denitrification (narG, nirK, and norB). Target genes primer names and concentrations (mM) and accession numbers for gBlocks (Integrated DNA Technology, UK) used to construct the standard curves are provided in Table S11. gBlocks were re‐suspended in Tris‐EDTA (10 mM Tris–HCl, 1 mM disodium EDTA, pH 8), following the manufacturer's instructions, adjusted to a final concentration of 10 ng/μL, and stored at −20°C until use. Linearized plasmids were used for the narG and norB genes as standards. PCR products of the correct size were gel‐extracted using the Nucleospin PCR Clean‐up Kit (Macherey‐Nagel, Germany) and cloned into *E. coli* JM109 cells using the pGEM‐T Easy vector system (Promega, USA). Clones were grown in TYP broth, and plasmids were extracted using the Nucleospin Plasmid EasyPure kit (Macherey‐Nagel, Germany). Sequencing of plasmids was conducted at HyLabs (Israel) using T7‐SP6 primers, and product identities were confirmed through BLAST searches. Plasmids containing target products were linearized with the SacI restriction enzyme, purified, and used for standard curve generation through serial dilutions to determine gene concentrations. The qPCR reactions were performed using the Fast SYBR Green Master Mix (Applied Biosystems, USA). The PCR reactions were carried out in a volume of 20 μL, containing 12.5 μL master Mix, primer concentration (between 0.15 and 0.5 mM) and 2 μL template DNA. PCR conditions included an activation step at 95°C for 20 s, followed by 40 cycles of denaturation at 95°C for 3 s and annealing/extension at 60°C for 30 s. During initial calibration, reactions were visualized on 1.5% agarose gels to confirm specificity and amplification of the correct band size. Melt curves were generated for all reactions to assess specificity, confirming a single amplicon by detecting a single distinct melting temperature peak.

### 2.7 16S rRNA Gene V4 Amplicon‐Sequencing

We performed 16S gene amplicon‐based sequencing, using the primer pair CS1_515F (ACACTGACGACATGGTTCTACAGTGCCAGCMGCCGCGGTAA) and CS2_806R (TACGGTAGCAGAGACTTGGTCTGGACTACHVGGGTWTCTAAT) (Sigma‐Aldrich, Israel) (Caporaso et al. 2012). The initial PCR was carried out in 25 μL reactions containing 12.5 μL of KAPA HiFi HotStart ReadyMix (KAPA Biosystems, Wilmington, WA, USA) and 0.75 μL of forward and reverse primers at a final concentration of 300 nM each. PCR conditions included an initial denaturation at 95°C for 3 min, followed by 30 cycles of 98°C for 20 s, 60°C for 15 s, and 72°C for 30 s. PCR products were visualized on a 2% agarose gel to assess band intensity. Samples were pooled and purified using calibrated Ampure XP beads before being used for library preparation. PCR visualization, purification, library preparation, and sequencing (2 × 250 bp paired‐end reads) were conducted on an Illumina MiSeq (at HyLabs, Israel). Demultiplexing of paired‐end reads and subsequent analyses were performed using QIIME2 (v2020.11) (Bolyen et al. 2019). Sequencing quality was assessed using the q2‐demux plugin, followed by chimera detection and merging of reads into Amplicon Sequence Variants (ASVs) using the q2‐dada2 plugin (Callahan et al. 2016). ASVs were defined by clustering at 100% similarity (Rognes et al. 2016) to account for length variations. Taxonomy was assigned using the SILVA 138 QIIME release database clustered at 99% similarity (Quast et al. 2012). The classifier was trained with the extract‐reads and fit‐classifier‐naive‐bayes methods via the q2‐feature‐classifier plugin (Bokulich et al. 2018), and ASV classification was performed using the classify‐sklearn method (ver. 0.23.1) (Pedregosa et al. 2011). The q2‐diversity plugin was used to generate rarefaction curves at varying depths, to confirm ASVs reached a plateau and to justify the chosen rarifying depth (Figure S4). Diversity analysis was also performed using the q2‐diversity plugin, at a rarifying depth of 16,159 ASVs. Alpha (Shannon's entropy, Pielou's evenness index and Faith's PD index) and beta (jaccard and bray‐curtis) metrics were constructed and presented in Supporting Information Results section 1.2. Venn diagrams were constructed based on the rarefied ASVs tables, obtained from the q2‐diversity plugin. Downstream analyses were conducted in R using the phyloseq (McMurdie and Holmes 2013) and ggplot2 (Wickham 2016) packages. Based on the 16S rRNA data, we performed a pathway prediction analysis using the PICRUSt2's (Phylogenetic Investigation of Communities by Reconstruction of Unobserved States) package (Douglas et al. 2020). The Kyoto Encyclopedia of Genes and Genomes (KEGG) database (Kanehisa and Goto 2000) was used to identify Orthology (KO) groups and modules (MO), related to the CH_4_ and nitrogen cycles (Data S6 and S7). Since 16S rRNA gene copy number variation introduces bias in relative abundance estimates, this bias propagates into functional predictions generated by PICRUSt2 (Gao and Wu 2023). To correct for this, we adjusted PICRUSt2 predictions using qPCR‐derived gene abundances, providing a more accurate representation of functional potential in the microbial community.

Raw sequence reads were deposited in the European Nucleotide Archive (ENA), EMBL European Bioinformatics Institute (EMBL‐EBI) Database BioProject accession number PRJEB59938 (https://www.ebi.ac.uk/ena/browser/home). Additional data are available under the Supporting Information sections.

### 2.8 Statistical Analysis

Statistical analyses were performed using the R (v. 4.0.3) rstatix package and the QIIME2 (v. 2020.11) software. For the PCA analysis, variables were log transformed, scaled, and centered to account for variations in magnitude and units. Statistical analysis for all microbiome diversity analysis was done using the rstatix package (Kruskal–Wallis and Dunn's tests) and beta‐group significance (PERMANOVA, PERMDISP, and ADONIS with 999 permutations). *p*‐values were adjusted according to the Benjamini–Hochberg FDR correction. All statistical tests were two‐sided. The *p*‐value was considered significant if < 0.05.

## 3 Results and Discussion

### 3.1 North Star Yedoma: A Warmer, More Advanced State of Talik Formation

Thermokarst mound development has been observed across the Pan‐Arctic yedoma regions (Walter Anthony et al. 2024); however, in the coldest, continuous permafrost areas, such as north Siberia, taliks have yet to form beneath yedoma thermokarst mounds (Beermann et al. 2017; Bottos et al. 2018; Hugelius et al. 2020; Marushchak et al. 2021; Nitzbon et al. 2020; Wegner et al. 2022; Weiss et al. 2016). Given the warm climate of interior Alaska (mean annual air temperature of −2.2°C ± 1.2°C; ACIS 2023) and advanced stage of talik development (5–9 m of thaw since 1978), NSY can provide insights about potential future directions related to greenhouse gas production, following permafrost thaw and talik formation in other yedoma subregions.

During summer at NSY, oxic conditions persisted down to 50 cm depth and temperatures gradually declined, from ~9°C (at 10 cm depth) to ~5.5°C (at 250 cm). In winter, soil‐surface (10 cm) temperature neared the freezing point (−0.42°C), gradually elevating to 1.09°C (250 cm) (Data S8). With soil‐surface freezing, anaerobic conditions at the top 15 cm (−1.22%) turned to semi‐aerated at 50 cm. Year‐round anaerobic conditions were measured at 100 cm and 190 cm (~−1.3%) (Data S8). Our previous study, which included modeling, remote sensing, geophysics, and field observations, showed that NSY represents a more advanced state of permafrost thaw and talik formation among upland yedoma landscapes (Walter Anthony et al. 2024), providing an opportunity to study these projected global warming effects. To the best of our knowledge, this study is the first to investigate the microbial community dynamics and function related to the CH_4_ and nitrogen cycles in upland yedoma taliks.

### 3.2 Seasonal Changes in Physico‐Chemical Parameters

**FIGURE 2:**
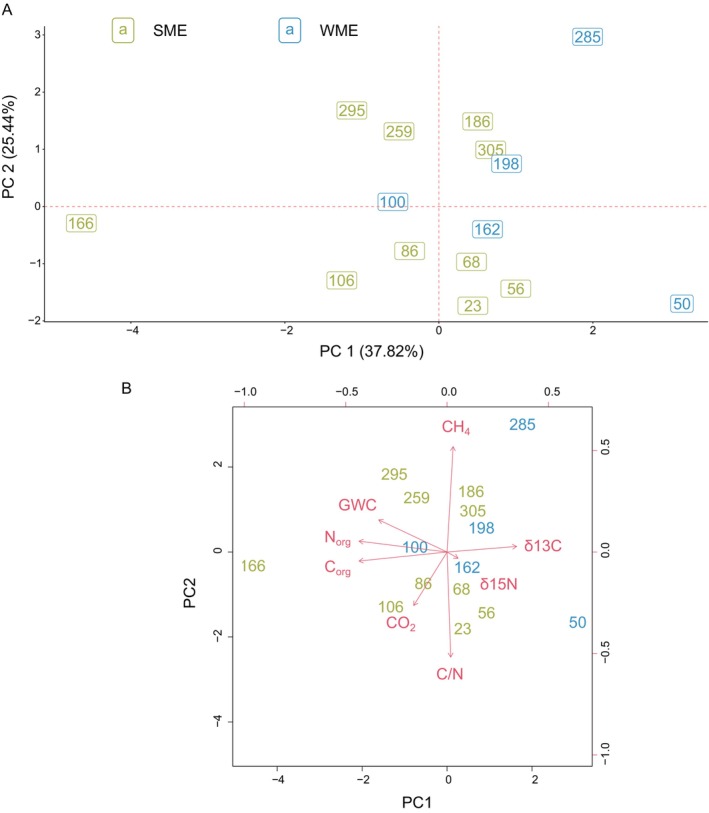
PCA analysis of selected environmental parameters during winter and summer at the NSY study site. (A) PCA analysis (*n* = 15) was performed on shallow summer (September 15, 2021) and winter (March 18, 2023) samples (up to ~3 m). SME = Summer Mid Elevation (id BH1, *n* = 10, olive‐green), and WME = Winter Mid Elevation (id BH6, *n* = 5, blue). (B) Loading scores, indicating the importance of tested environmental variables related to PC 1 and PC 2.

**FIGURE 3:**
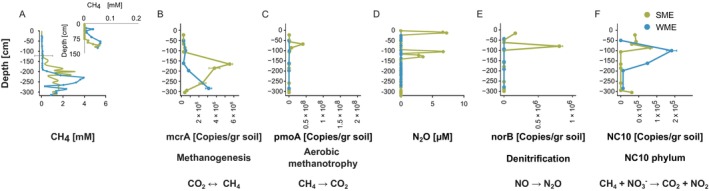
Absolute abundance soil depth profiles of CH4, N_2_O concentrations, and genes related to the nitrogen and CH4 cycles. Quantification of absolute abundances (*n* = 15 samples) was performed on shallow summer (September 15, 2021) and winter (March 18, 2023) samples (up to ~3 m), using primers targeting functional genes related to the nitrogen and CH4 cycles (see Table S11 for a detailed list of genes and primers). The absolute abundance of each gene is presented as copies/gr soil (mean ± SE). (A) CH4 concentrations in summer and winter boreholes depth profiles. (B‐C) mcrA and pmoA gene expression (D). N_2_O concentrations in summer and winter boreholes depth profiles. (E) norB gene expression and (F) NC10 phylum gene expression. For each gene, the related function and main reaction (main products, unbalanced) are indicated. The double arrow symbol (↔) represents potential forward and reverse methanogenesis (mcrA gene). Nitrous oxide and CH4 concentrations are also presented. SME = Summer Mid Elevation (ID: BH1, *n* = 10, olive‐green), WME = Winter Mid Elevation (BH6, *n* = 5, blue).

Our PCA analysis of summer and winter samples ≤ 3 m depth showed distinct seasonal clustering of physico‐chemical parameters (PC1‐PC2: 37.82%–25.44%, *n* = 15, Figure 2A,B and Table S1). Summer samples (SME) clustered into two groups (23–106 cm and 186–305 cm) with shallower samples presenting elevated CO_2_ concentrations and C/N ratios, and lower CH_4_ concentrations (i.e., production) (Figure 2B and Table S1). This clustering into distinct depth‐based groups likely reflects stratified microbial processes, driven by variations in oxygen availability and organic matter availability. These results are consistent with previous reports indicating that active aerobic microbial decomposition is driven by organic matter and O_2_ availability (Müller et al. 2018). In comparison, winter samples (WME) showed substantial variability. Although during both seasons, CH_4_ production increased with depth (peaking > 200 cm belowground), near the ground surface CH_4_ concentrations were only observed in winter (Figure 3A and Table S1). This is consistent with our previous report that winter CH_4_ emissions to the atmosphere are three times higher compared to summer (Walter Anthony et al. 2024). The C_org_/N_org_ ratios were low in both seasons (SME: 13 ± 0.45, WME: 12.42 ± 0.69; Table S1), supporting the existence of a high N stock (Marushchak et al. 2021; Strauss et al. 2015) and potential increases in N_2_O production (Wegner et al. 2022). Two peaks in N_2_O concentrations were observed only in the summer profile (10 cm, 7.2 μM; and 105 cm, 6.7 μmole/L; Figure 3D).

The counter behavior of CH_4_ and N_2_O raises significant concerns, as N_2_O is about nine times more efficient in trapping heat than CH_4_ (Stocker et al. 2013). During AOM by *Methylomirabilis Oxifera*, nitrite is reduced and used as an electron acceptor. By NO_2_^−^ labeling experiment, Ettwig et al. (2010) (Ettwig et al. 2010) found that while most of the culture reduced NO_2_^−^ to N2, 7% was reduced to N_2_O by other denitrifiers. A similar ratio during denitrification in favor of N_2_O was also observed in thawed loamy soils (Tenuta and Sparling 2011). From mass balance calculation, for 30 CH_4_ molecules that are anaerobically oxidized, one molecule of N_2_O is released. This will reduce CH_4_ sink of AOM by as much as 30% (as N_2_O is nine times more potent then CH_4_ as a GHG).

### 3.3 Seasonal Shifts in Methanogenesis and Methanotrophy

Patterns of absolute gene expression [mcrA (methanogenesis) and pmoA (methanotrophy)] were consistent with CH_4_ concentration profiles in both seasons (Walter Anthony et al. 2024). While higher mcrA levels were measured in summer across most anaerobic depths, peaking at 166 cm (Figure 3B and Table S5), winter levels peaked at a greater depths (285 cm). Close to the soil surface, summer methanotrophy was high (68 cm), diminishing by 30‐fold in wintertime (Figure 3C and Table S5), probably due to inhibition related to the near 0°C temperatures (Walter Anthony et al. 2024). Indeed, elevated temperatures enhance the expression of both genes (Liebner et al. 2015; Wang et al. 2020; Wei et al. 2018). As in the upland yedoma domain, similar depth‐related patterns in pmoA and mcrA gene expression have been reported in the Arctic and discontinuous permafrost‐affected regions (Barbier et al. 2012; Liebner et al. 2015; Tang et al. 2023). While higher mcrA levels are typically reported at deeper anaerobic horizons, pmoA expression peaks closer to the soil surface.

**FIGURE 4:**
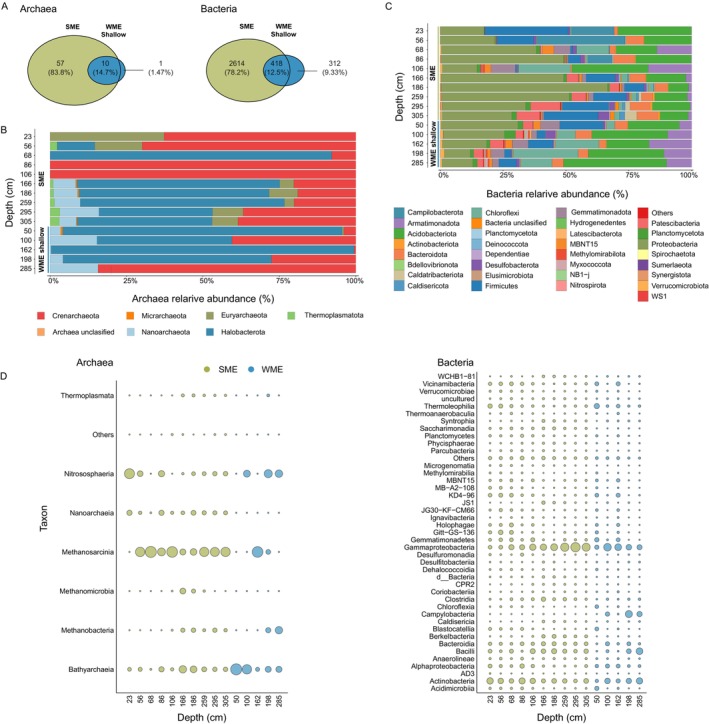
Shifts in microbial community composition at North Star Yedoma. Analysis of community composition was based on 16S rRNA gene amplicon‐based sequencing. Three cores were included in the analysis. SME = Summer Mid Elevation (ID: BH1, *n* = 10, olive‐green), WME = Winter Mid Elevation (BH6, *n* = 5, blue). (A) Venn diagram of archaea (left panel) and bacteria (right panel) representing the number of shared and unique ASVs. (B + C) Relative abundance barplot visualization of archaeal (B) and bacterial (C). ASVs (minimum frequency ≥ 1%). Samples are ordered on the Y axis according to borehole and depth. (D) Bubble plot representing class level relative abundances of archaea (left panel) and bacteria (right panel). Samples are ordered on the X axis according to borehole and depth. Bubble size indicates relative abundance.

To better understand the seasonal differences in gene expression, we assessed the microbial community composition. Our analysis revealed a diverse microbial community in both seasons, with genera present at low relative abundance (< 1%, Data S1 and S2). Relatively high microbial diversity has been previously reported in different permafrost environments, with notable variation across distinct permafrost types and geographic locations (Jansson and Taş 2014). Overall, the microbial diversity in our study was higher in summer, with about five times more unique archaeal and bacterial Amplicon Sequence Variants (ASVs) compared to winter (Figure 4A). Alterations in microbial community related to seasonality and permafrost thaw have also been previously reported in both phylogenetic identity and functional gene abundances (MacKelprang et al. 2011). The dominant archaea were classified as *Halobacterota* and *Crenarchaeota* (Figure 4B,D and Data S3). The prevalent bacterial taxa included *Proteobacteria* (10%–32%, mainly *Gammaproteobacteria*), *Actinobacteria* (20%–25%), followed by *Firmicutes*, *Chloroflexi*, and *Bacteroidota* (Figure 4C,D and Data S4). These results are consistent with reports of microbial communities identified in previous permafrost studies (Hultman et al. 2015; Jansson and Taş 2014; MacKelprang et al. 2011, 2017; Steven et al. 2008; Yergeau et al. 2010), suggesting these taxa are common across diverse permafrost environments.

A detailed description of seasonal shifts related to alpha and beta diversity analyses (section 1.1) and microbial community composition (section 1.2) is presented in the Supporting Information Results section.

**FIGURE 5:**
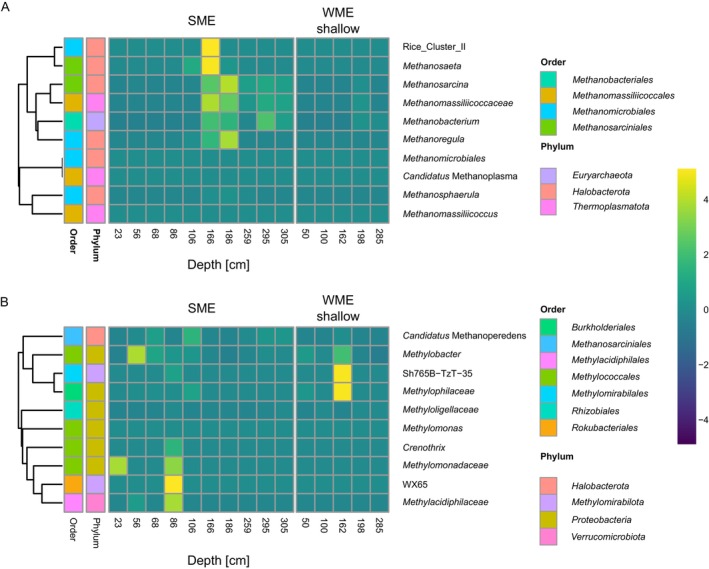
Shifts in the methanogenic and methanotrophic microbial communities at North Star Yedoma. Analysis of microbial community composition was based on 16S rRNA gene amplicon‐based sequencing. Heatmap visualizations present relative abundance of methanogenic (A) and methanotrophic (B) taxa. Order and Phylum are presented to the left of the heatmaps. Samples (columns) were ordered according to depth and faceted according to sampling environment. SME = Summer Mid Elevation (ID: BH1, *n* = 10, olive‐ green), WME = Winter Mid Elevation (BH6, *n* = 5, blue). The color scale represents relative abundance of ASVs within each sample, ranging from low (blue to black) to high (yellow to green).

During summer, a diverse methanogenic community was detected mainly between 166 and 186 cm depths (Figure 5A and Data S5) that corresponded to the CH_4_ levels and gene expression profiles. This included obligately acetoclastic (*Methanosaeta*) (Carr et al. 2018), hydrogenotrophic (*Methanobacterium* and *Methanoregula*) (McCalley et al. 2014), and methylotrophic methanogens (e.g., Methanomassiliicoccaceae) (Narrowe et al. 2019) as well as versatile mixotrophs (*Methanosarcina*) (Lackner et al. 2018), representing all forms of methanogenesis. Furthermore, such stratification aligns with studies of permafrost systems, where intermediate depths act as methane production hotspots due to converging anoxia and labile carbon availability (Freitas et al. 2025; Gerera et al. 2025). PICRUSt2's KEGG pathway prediction analysis corroborated these results, indicating three pathways of methanogenesis (Figure S8A and Data S6 and S7). In comparison, although winter CH_4_ concentration and mcrA levels peaked at 285 cm, methanogens were only identified at very low relative abundance (Figure 5A and Data S5–S7). A possible explanation for this discrepancy is their presence in soil layers adjacent to this depth that were not sampled.

During summer, in anaerobic horizons corresponding to and overlying the methanogenic zone, N‐AOM coupled to NO_3_^−^ and NO_2_^−^ reduction was exemplified by the high prevalence of the anaerobic methanotroph archaeon *Candidatus* Methanoperedens (ANME2d) (Figure 5B and Data S5). Very high relative abundance was noted at 106 cm (5.5% of), corresponding to the identified N_2_O peak (Figure 3D), and at 68 cm (5.9%). The high relative abundance at 68 cm is surprising given the semi‐aerated conditions (Data S8). However, given that the oxygen probes were located at specific depths, at thermokarst‐mound flanks adjacent to the EC tower in the vicinity of the boreholes, it is likely these did not fully reflect the oxygen concentrations. High ANME2d relative abundance suggests significant N‐AOM in this environment. In contrast, a significant seasonal shift was noted during winter, as ANME2d relative abundance plummeted to very low levels. ANME representatives, including *Candidatus* Methanoperedens, have been previously reported in permafrost environments (Ren et al. 2022; Winkel et al. 2018, 2019). Coupling AOM to NO_3_^−^ / Fe^3+^ reduction (Ettwig et al. 2016), they can perform reverse methanogenesis via the mcrA gene and are suggested as potential mitigators of CH_4_ production and subsequent emissions to the atmosphere (Haroon et al. 2013; Yang et al. 2022). Yet, concurrently, *Candidatus* Methanoperedens has been related to direct increases in N_2_O generation (Tan et al. 2024). We detected at this depth (and beyond) high relative abundance of the genus *Pseudomonas* (7.1% at 86 cm, Data S2). Members of this genus can perform complete aerobic denitrification and were shown to possess genes catalyzing the reduction of N_2_O to N_2_ (Ji et al. 2015; Wunsch and Zumft 2005; Yang et al. 2020). *Pseudomonas* can also oxidize CH_4_ and perform AOM with NO_3_^−^ and NO_2_^−^ (Ferenci et al. 1975; Pang et al. 2021). The latter, together with increased N availability, was previously reported following permafrost thaw, culminating in elevated N_2_O emissions to the atmosphere (Cui et al. 2018; Treat et al. 2016; Yang, Peng, Olefeldt, et al. 2018).

Just below the ANME2d maximum peak, we also identify via qPCR the presence of the NC10 phylum, at similar levels in both seasons (Figure 3F). We identified the genus Sh765B‐TzT‐35 (*Methylomirabilaceae*) in the microbial community analysis, albeit at low relative abundance (Figure 5B and Data S5). Members of this family are known to perform aerobic CH_4_‐oxidation (via an intra‐aerobic pathway) under anaerobic conditions, coupled to NO_2_^−^ reduction (Ettwig et al. 2010). The NC10 phylum has been previously detected in permafrost peatlands, in mid‐soil layers where redox gradients and nitrogen availability supported their activity (Fu et al. 2025). This highlights their potential role in influencing biogeochemical processes in climate‐sensitive systems. Microorganisms like ANME2d and NC10 oxidize CH_4_, but at the same time, N_2_O may be generated through interactions with other members of the microbial consortia (Ettwig et al. 2010). Shifts in microbial community dynamics (e.g., community composition and spatial distribution) of methanogens, methanotrophs, and microorganisms related to the nitrogen cycle have also been previously reported and associated with changes in environmental conditions such as temperature, pH, water content, soil structure, oxygen level, nutrient availability, carbon and nitrogen content, thaw depth, and permafrost collapse (Barbier et al. 2012; Feng et al. 2020; Holm et al. 2020; Hultman et al. 2015; Jansson and Taş 2014; Ji et al. 2020; MacKelprang et al. 2017; Monteux et al. 2020; Singleton et al. 2018; Song et al. 2020; Tang et al. 2023; Waldrop et al. 2023; Wang et al. 2020; Yuan et al. 2018).

### 3.4 Seasonal Shifts in Genes Related to Denitrification and Ammonium Oxidation

N‐AOM was observed at and above the methanogenic zone, predominantly during summer, indicating couplings between the CH_4_ and nitrogen cycles. To examine the potential for N_2_O production, we measured norB absolute gene expression. Similar to the measured N_2_O concentrations, we identified two norB peaks at the top 1 m, only during summer (Figure 3E and Table S5). These observations raise concerns regarding elevations in N_2_O production and potential emissions to the atmosphere, as previously reported from thermokarst mounds in North Siberian yedoma permafrost (Marushchak et al. 2021). However, unlike NSY with its extended talik formation, North Siberia soils are thawed only several centimeters deep during summer and lack taliks entirely.

Additional nitrogen‐cycle genes showed similar absolute expression patterns, with elevated levels closer to the soil surface in summer (up to 86 cm) (Figure S2A–F and Table S5). Thereafter, absolute abundance levels dropped sharply. These results indicated processes related to denitrification (narG and nirK genes) and anaerobic ammonium (NH_4_^+^) oxidation to N_2_ (Anammox, hzsB) and to NO_2_^−^ (Feammox: 16S of *Acidimicrobiaceae* sp. strain A6). Below these semi‐aerated depths, aerobic oxidation of NH_4_^+^ to NO_2_^−^ by bacteria and archaea (amoA gene) peaked between 23 and 56 cm depths. During winter, expression was low for nearly all genes, with slight elevations between 100 and 162 cm. Pathway prediction analysis related to the nitrogen cycle also indicated elevated levels near the surface for the summer samples (up to 86 cm depth; Figure S8B and Data S6 and S7). This included nitrification, denitrification, dissimilatory nitrate reduction, and complete nitrification, comammox. Contrary, winter nitrogen‐related pathways were predicted for the most part at very low levels, with slight elevations at deeper sediment depths (~162 cm). Winter denitrification was predicted at relatively high levels as during summer.

Our data indicate significant seasonal shifts in microbial community dynamics and function, related to both the CH_4_ and nitrogen cycles. During summer, we observed N‐AOM, together with elevated N_2_O production in the interior Alaska upland yedoma talik. Global warming is projected to drive widespread permafrost thaw and talik formation across the entire yedoma domain, mobilizing vast pools of old carbon and nitrogen (Monteux et al. 2020; Strauss et al. 2022). These can become accessible to microbial mineralization and stimulation of GHG emissions to the atmosphere, including N_2_O (Holm et al. 2020; Monteux et al. 2020; Wegner et al. 2022). The latter has been related to the activity of denitrifying microorganisms in shallow, surface soils (Altshuler et al. 2019; Marushchak et al. 2021; Yang, Peng, Marushchak, et al. 2018). To date, most studies represent continuous‐permafrost or non‐yedoma environments. Augmented permafrost thaw can alter soil temperature, water retention, drainage, and soil moisture (Walvoord and Kurylyk 2016), in a way that is difficult to predict on a large‐scale basis. Our NSY upland study site is characterized by relatively dry soil conditions, with GWC ranging between 18% and 32% (Table S1 and Walter Anthony et al. 2024). Previous reports indicate intermediate moisture content promotes N_2_O emissions to the atmosphere from yedoma and other permafrost‐affected soils (Hugelius et al. 2020; Strauss et al. 2024). Thus, elevated GWC in upland yedoma areas of continuous‐permafrost may facilitate the establishment of denitrifying and N_2_O producing microbial communities that may augment GHG emissions (Marushchak et al. 2021). With recent data suggesting that nitrogen mineralization and turnover rates in permafrost‐affected active layers are comparable to those in temperate and tropical soils (Ramm et al. 2022), our findings underscore the importance of microbial and biogeochemical dynamics related to widespread permafrost degradation and talik formation across the yedoma domain. These seasonal shifts suggest that temperature‐associated changes in permafrost thaw may influence microbial activity, with a possible link to greenhouse gas (GHG) emissions.

### 3.5 Depth Profile of the Full Talik Down to the Top of Permafrost

The yedoma domain, though small in area, contains a large share of the northern permafrost soil C_org_ and N_org_ pools extending tens of meters deep (Hugelius et al. 2014; Strauss et al. 2022). Heat transport within the thawing yedoma permafrost further accelerates talik formation, facilitating microbially mediated organic matter mineralization (Walter Anthony et al. 2024). Analyzing the depth profile of the full talik down to the top of permafrost at NSY provides an opportunity to study CH_4_ and nitrogen‐related microbial processes linked to GHG emissions, particularly in the context of global warming and talik formation.

We analyzed a full‐talik‐profile winter core of ~7 m and performed a separate PCA analysis of the winter's shallow (< 3 m) versus deep (> 3 m) samples. Clear clustering of the two groups was noted, with great variability in the shallow samples (Figure S1 and Table S4). Deeper samples were characterized by higher CO_2_ and lower C_org_ and N_org_ levels (Figure S1 and Table S4). An additional peak in CH_4_ concentrations was measured at deeper layers (535–655 cm). This was accompanied by elevated mcrA expression at 711 cm. This offset at the deeper layers may be related to upward gas migration (Walter Anthony et al. 2024). The absolute expression levels of all other genes related to aerobic methanotrophy and the nitrogen cycle were low across all deeper depths (Figure S3A–L and Table S5). In addition, we did not identify any N_2_O production.

Deep samples presented a higher proportion of overall unique archaea compared to shallower samples (56% vs. 28%; Figure S5A). Correspondingly, the methanogenic community was more diverse and detected at a higher relative abundance (Figure S6A and Data S5). As with the shallow samples, pathway prediction analysis at deeper depths supported mainly acetoclastic methanogenesis (Figure S8A and Data S6 and S7). The aerobic methanotrophic community was less diverse and included only members of the *Methyloligellaceae* family (Figure S6B and Data S5, see below for elaboration). The methanotrophy pathway prediction was very low (Figure S8A). Additional changes in the bacterial and archaeal community composition were also observed (Figure S5B–D, Data S4). A detailed description of the microbial community composition of both groups is presented in Supporting Information Results 1.3.

Our results indicate significant differences in microbial community dynamics, between shallow and deeper depths of the winter full talik profile. The diminished CH_4_ and nitrogen‐related processes at the deeper talik horizons, compared to the soil surface, may be related to functional constraints, leading to decreased N‐AOM, higher CH_4_ emissions, and lower N_2_O production. Microbial communities at deeper, more ancient yedoma permafrost deposits may be shaped by long‐term cryogenic conditions, exhibiting specialized survival strategies for persistence in the frozen, isolated, resource‐limited environment (MacKelprang et al. 2017). Additional constraints on carbon and nitrogen cycling in these ecosystems have been attributed to missing microbial functions (Monteux et al. 2020). While freshly thawed wet yedoma sediments exhibit low N_2_O emissions due to such limitations on key microbial functional groups, following long‐term thaw, significant shifts can alter microbial community composition and function, resulting in higher N_2_O emissions (Marushchak et al. 2021).

### 3.6 Elevation and Aeration Driven Alterations in Microbial Dynamics in the CH_4_ and Nitrogen Cycles

During summer, we analyzed two boreholes, characterized by high (SHE, *n* = 6, up to 225 cm) and mid elevation (SME, *n* = 10, up to 305 cm), corresponding to deep versus shallow aeration conditions. Lower CH_4_ concentrations were measured throughout the high elevation core, peaking at 225 cm (0.06 mM, Figure S3A and Table S1). Corresponding patterns were noted in the mcrA and pmoA gene absolute expression (Figure S3B,C and Table S5), supporting the existence of a deeper aerobic zone. pmoA peaked near the soil surface (SHM, 8 cm), while CH_4_ was absent at top‐soil aerated layers, as in SME samples. In agreement, methanogens were absent from the SHE borehole (Figure S6A and Data S5). These results, together with the relatively low CH_4_ production and positive emissions to the atmosphere in chamber fluxes (Walter Anthony et al. 2024), suggest that a methanogenic community is likely present at deeper layers. A detailed description of the microbial community composition (Figure S5B–D, Data S4) is presented at Supporting Information Results 1.4.

One relatively low N_2_O peak was noted in the high elevation samples (2.7 μM at 91 cm, Figure S3D), and norB expression in SHE was below detection limit at all depths (Figure S3E). These differences may be related to increased N availability at lower altitudes (Sousa Neto et al. 2011; Teh et al. 2014), or to other contributing factors indirectly related to elevation, including temperature, soil and water chemistry, oxygen availability, and microbial activity (Davidson et al. 2000; Fatumah et al. 2019). Nitrogen‐cycle related genes of the SHE samples followed a similar expression pattern to that of SME, peaking closer to the soil surface (Figure S3F–L and Table S5), followed by sharp declines.

Interestingly, we identified very high relative abundances of *Candidatus* Methanoperedens in the SHE core, at 146 cm (9.2% of all ASVs) and 195 cm (5.9%) depths (Figure S6B and Data S5; discussed below).

### 3.7 Methane Dynamics in Upland Yedoma Soils Affected by Permafrost Thaw and Talik Formation

**FIGURE 6:**
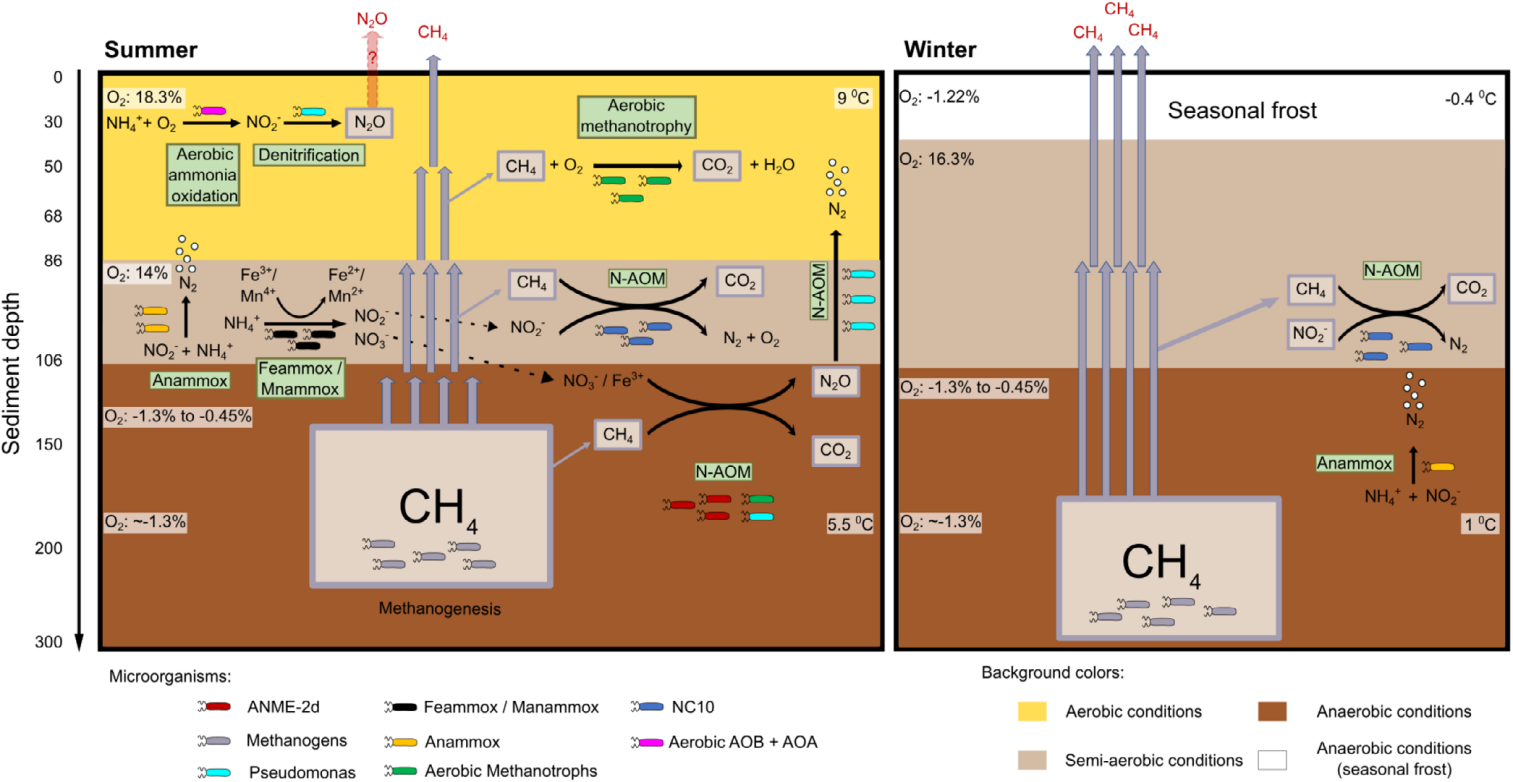
Methane dynamics in upland yedoma, suggested mechanisms. During summer (left panel), CH4 is oxidized by both anaerobic (at deeper) and aerobic (at shallower layer) methanotrophs (i.e., ANME2d, NC10, and denitrifiers performing N‐AOM). Methane oxidation is coupled to the nitrogen cycle, with N‐AOM linking CH4 and nitrogen transformations. N_2_O is produced and present up to 1 m depth. In winter, near‐freezing temperatures at the soil surface limit microbial activity and methanotrophy. The diminished CH4 oxidation and nitrogen‐related processes at deeper horizons may be related to lower temperatures, functional constraints, or nitrogen availability, leading to decreased N‐AOM, higher CH4 emissions to the atmosphere, and lower N_2_O production. The suggested mechanisms are based on chemical and qPCR profiles, and 16S amplicon‐based sequencing analyses. The background colors represent different soil oxygenation conditions (see Materials and Methods and Data S8) (Walter Anthony et al. 2024).

We previously reported higher methane fluxes measured in the winter borehole, compared to summer (165.8 ± 4.0 vs. 55.7 ± 2.3 mg CH4 m^−2^ d^−1^, mean ± SEM) (Walter Anthony et al. 2024). Focusing on seasonal variations, the current study delineates the spatio‐temporal dynamics of methanogenic and methanotrophic microbial communities in the upland yedoma. Our findings highlight the importance of couplings between methanotrophy and the nitrogen cycle, and their potential implications for GHG generation. Elevated summer methanotrophy and N_2_O production coincided with higher relative abundances of aerobic and anaerobic methanotrophs (i.e., ANME2d, NC10 and denitrifiers performing N‐AOM). Given that N_2_O is approximately nine times more potent as a GHG than CH_4_, the contribution of this surface‐associated production to overall emissions to the atmosphere is expected to be significant. During winter, near‐freeze temperatures at the soil surface limited microbial activity and methanotrophy (Figure 6 and Data S8). N‐AOM was reduced, and ANME2d were largely absent, possibly due to reduced nitrogen availability. We did not observe N_2_O generation in winter, a time when CH_4_ emissions were high.

These mechanisms demonstrate the dynamic interplay between the CH_4_ and nitrogen cycles and are illustrated in Figure 6, based on the chemical, qPCR, and 16S amplicon‐based sequencing analyses.

Microbial communities in permafrost soils are important players in the regulation of carbon and nitrogen cycling. Thawing of yedoma permafrost may liberate significant nitrogen stocks that are predicted to contribute to denitrification and N‐AOM, potentially elevating N_2_O production and subsequent emissions to the atmosphere (Marushchak et al. 2021; Strauss et al. 2022, 2024; Wegner et al. 2022). GHGs are produced by microbial activity that is strongly influenced by the community composition and its functional capacities (Hugelius et al. 2020; Monteux et al. 2020). The microbial processes in yedoma soils are often limited by the functional constraints imposed by prolonged freezing over millennia. Thawing may alleviate some of these constraints by introducing novel—functionally distinct microbial communities, facilitating carbon and nitrogen cycling (Monteux et al. 2020; Wegner et al. 2022) and augmenting aerobic and anaerobic methanotrophy at deeper layers, including N‐AOM. These are influenced by factors like moisture, oxygen availability, and temperature (Marushchak et al. 2021; Voigt et al. 2020). Permafrost carbon and nitrogen feedbacks/interactions/couplings in yedoma soils, and the role of nitrogen cycling, particularly processes like N‐AOM, remain poorly understood (Marushchak et al. 2021; Strauss et al. 2024).

## 4 Concluding Remarks

The interplay between permafrost thaw, mobilization of carbon and nitrogen stocks, microbial activity, and elevated GHG emissions accentuates the significance of yedoma permafrost soils. Yet, most studies conducted on yedoma permafrost focused on the uppermost layers of uplands in the colder, continuous permafrost zone where taliks have not yet been documented or on thermokarst lakes. Our data, together with previous flux compilations, show that during both summer and winter, upland yedoma taliks are a surprisingly high source for CH_4_ and potential for N_2_O emissions. Our soil analyses suggest that emissions of N_2_O, unlike CH_4_, may be higher in summer than in winter. Unsaturated‐thawed yedoma permafrost harbors a diverse microbial community, with distinct spatio‐temporal shifts in composition and function. This is evident in the methanogenic and methanotrophic community dynamics, as well as N‐AOM processes. These findings are particularly important under continued global warming, which is expected to cause widespread talik formation in the colder yedoma region of North Siberia by the end of this century and warmer upland yedoma soils everywhere.

## Author Contributions

**Oded Bergman:** conceptualization, data curation, formal analysis, software, validation, writing – original draft, writing – review and editing. **Katey Walter Anthony:** conceptualization, funding acquisition, investigation, methodology, supervision, writing – review and editing. **E. Eliani‐Russak:** data curation, investigation, methodology. **Orit Sivan:** conceptualization, funding acquisition, methodology, project administration, supervision, writing – review and editing.

## Conflicts of Interest

The authors declare no conflicts of interest.

## Supporting information


Data S1.

## Data Availability

The data that support the findings of this study are openly available in Zenodo at https://doi.org/10.5281/zenodo.15766199and Github at https://github.com/BergmanOded/Yedoma_GCB2025. Raw sequence reads are openly available in the European Nucleotide Archive (ENA), EMBL European Bioinformatics Institute (EMBL‐EBI) Database BioProject accession number PRJEB59938.
