# Supplementary material for: Nitrogen Redox Controls on Greenhouse Gas Production in Yedoma Taliks
Source: Glob Chang Biol. 2025 Jul 21;31(7):e70356. doi: 10.1111/gcb.70356 (PMC12277943; doi:10.1111/gcb.70356)
Supplement: Supplementary file 1 — Data S1. [file GCB-31-e70356-s001.zip › gcb70356-sup-0010-Supinfo.pdf]

Supplementary Information

Complex nitrogen redox couplings control methane and nitrous oxide production in Arctic upland yedoma taliks

Oded Bergman[1,2,*], Katey Walter Anthony[1], E. Eliani-Russak[2], Orit Sivan[2]

Affiliations

[1] Water and Environmental Research Center, University Alaska Fairbanks, Fairbanks, Alaska USA

[2] Department of Earth and Environmental Sciences, Ben Gurion University of the Negev, Beersheva, Israel

* Corresponding author: bergmano@bgu.ac.il. The Department of Geological and Environmental Sciences, Ben Gurion University of the Negev, David Ben Gurion Blvd 1, Beer Sheva, Israel.

The following are included:

Supplementary results

Supplementary Figs 1-8

Supplementary Tables 1-11

Supplementary Results

1. Microbial community composition

1.1 Seasonal alterations in the microbial community alpha and beta diversity

To further characterize the microbial community dynamics, we assessed alpha and beta diversity in all environments. Despite the occurrence of significantly more unique SME samples (Fig. 4A), Shannon's entropy, which accounts for both species richness and evenness, did not differ between the environments (Fig. S7A, Table S6). Pielou's evenness index was on the verge of significance (W = 6.9, $p = 0.074$, Table S6), indicating some variation in distribution of the microbial community - as is evident in the Phylum level barplots (Fig. 4B-C). Significant differences were noted in Faith's PD index (W = 19.18, $p = 0.0002$), which was higher for SME compared to shallow WME samples (Z = -2.936, $p = 0.007$). In all, Higher phylogenetic diversity was observed during summer compared to winter samples (Fig. S7A, Table S6 and S7).

Beta diversity differed for both Jaccard (PC1-PC2: 13.98%-9.2%; PERMANOVA: $F_{4,27}$=2.4, $p=0.001$) and Bray-Curtis metrics (PC1-PC2: 20.13%-13.73%; PERMANOVA: $F_{4,27}$=3.79, $p=0.001$, Fig. S7B and Table S8). PERMDISP was not significant. Post hoc pairwise comparison analysis indicated all groups differed from one another (all tests are presented in Table S9). To determine which variables contributed to the observed differences in variance, we performed ADONIS tests, with the following: GMC, CH_4, CO_2, C_{org} and sampling environment. For both Jaccard and Bray-Curtis, the sampling environment was the top explanatory variable (R^2=0.19, $p=0.001$ and R^2=0.25, $p=0.001$; Table S10), followed by methane levels (R^2=0.084, $p=0.001$ and R^2=0.1, $p=0.001$) and GWC (R^2=0.06, $p=0.003$ and R^2=0.04, $p=0.001$). Overall, 41.3% of the variance explained by the Jaccard index and 51% by Bray-Curtis (Table S10). Additional relevant environmental variables such as temperature oxygen and pH, were not available per sample, and thus not included in the analysis.

1.2 Seasonal shifts in microbial community composition

Analysis of the microbial community composition, indicated summer samples contained five times more unique archaeal and bacterial ASVs compared to winter (Fig. 4A). Near the soil-surface, summer ASVs were dominated by *Crenarchaeota* and to a lesser extent by *Nanoarchaeota*, both significantly diminishing with depth as *Halobacterota* increased. Seasonal shifts were noted as winter samples at all depths were mainly *Crenarchaeota* (Fig. 4B). Importantly, we identified ASVs classified as the anaerobic archaeon methanotroph *Candidatus* Methanoperedens (ANME-2d), at high relative abundance, during summer, while being largely absent in winter, except at 162 cm depth (Fig. 5B and Supplementary Data 5).

Summer depth dependent alterations in bacterial phyla composition were observed, mainly between samples located closer to the soil-surface (up to 86 cm), and at deeper layers (between 106-305 cm; Fig. 4B and Supplementary Data 3). For example, the relative abundance of *Proteobacteria* increased significantly with depth, peaking at 295 cm. In contrast, *Chloroflexi*, prevalent in samples up to 86 cm depth, decreased in relative abundance with depth alongside an increase in *Firmicutes* ASVs (Fig. 4C and Supplementary Data 4). Seasonal trends were evident, with *Proteobacteria* diminished with depth during winter, contrary to their summer distribution. Opposite trends were observed for *Actinobacteria* and *Campilobacterota*.

1.3 Microbial community dynamics of the full talik down to the top of permafrost

Examination of the microbial community showed that shallow and deep winter (WME) samples presented similar numbers unique bacterial ASVs (48.5%-42.7%). Although the majority of unique archaeal ASVs were identified in the shallower samples (56% vs. 28%), count number was low (Fig. S5A). The dominated archaea in both environments were classified as the *Crenarchaeota* phylum (52% and 90%, respectively; Fig. S5B, D and Supplementary Data 3), at almost all depths. While the methanogenic community in shallower depths mainly consisted of *Methanobacterium*, in deeper depths a diverse community was noted (Fig. S6A and Supplementary Data 5), characterized mainly by: *Methanomicrobiales*, *Methanoregula*, *Methanobacterium*, *Methanosphaerula*, *Candidatus* Methanoplasma and *Methanomassiliicoccus*. The identity of these methanogens indicates a shift from acetoclastic methanogenesis in the shallower samples, to methylotrophic and hydrogenotrophic methanogenesis. However, this was not evident in the pathway prediction analysis (Fig. S8A and Supplementry Data 6-7). The methanotrophic

community throughout was much less diverse (Figs. S6B and Supplementary Data 5, see below for elaboration) and the aerobic CH_4 oxidation pathway prediction was very low (Fig. S8B and Supplementary Data 6-7).

The bacterial community composition significantly altered with depth. The percentages describing phyla abundances, denote prevalence throughout the borehole. In shallow samples, in the bacterial community *Proteobacteria* (28%), *Chloroflexi* (7%), and *Actinobacteria* (23%) were more prevalent. *Proteobacteria*, *Chloroflexi* diminished with depth as *Campilobacterota* (12%) and *Firmicutes* (10%) abundance increased, contributing to the community structure (Figs. S5C-D and Supplementary Data 3). The community composition in the WDLE samples is markedly different, with *Proteobacteria* dominate between 345-535 cm (23%), as well as *Actinobacteria* (23%), and *Firmicutes* (22%) to a lesser extent. Below 535 cm, community composition remained relatively similar and was comprised of *Proteobacteria* at lower relative abundance, with *Firmicutes* and *Actinobacteria* levels increasing. *Patescibacteria* (6%) and *Bacteroidota* (10%) were also more prevalent at these depths. Notably, Chloroflexi, which are prominent in shallow sammples, were significantly reduced (1% throughout the borehole) (Figs. S5C-D and Supplementary Data 3). The shift in community composition between shallow and deep samples highlights the influence of depth-related environmental gradients, including reduced oxygen availability and changes in nutrient profiles, shaping the microbial communities in deeper permafrost talik layers.

1.4 Elevation and aeration driven shifts in microbial community dynamics

Close to the soil surface, the archaeal community composition was, for the large part, similar between the two environments. ASVs were dominated by *Crenarchaeota* and to a lesser extent by *Nanoarchaeota*, both significantly diminishing with depth. Thereafter, the patterns exhibited in the SHE (from 146 up to 225 cm), was similar to that of the SME samples (68 up to cm 106 cm). Beyond this depth (from 166-305 cm), increases in the relative abundance of methanogens was noted in the SME samples, mainly of the phyla *Thermoplasmatota* (family *Methanomassiliicoccaceae*) and *Euryarchaeota* (genus *Methanobacterium*) (Figs. S5D and S6A-B, Supplementary Data 5). The methanogenic community was predominantly composed of acetoclastic methanogens (such as *Methanosaeta* and *Methanosarcina*), as well as hydrogenotrophic methanogens (*Methanoregula* and *Methanobacterium*)- between 106-305 cm depth (Figs. S5D and S6A-B, Supplementary Data 5).

Depth dependent alterations also were noted in bacterial phyla composition of SME, mainly between samples close to the soil-surface (up to 86 cm), to deeper samples (between 106-305 cm; Fig. S5B and Supplementary Data 3). For example, the relative abundance of *Proteobacteria* increased significantly with depth (peaking at 8 cm). *Chloroflexi* which were prevalent up to 86 cm depth, decreased in relative abundance alongside an increase in *Firmicutes* ASVs (Fig. S5C and Supplementary Data 4). For the SHE borehole, bacterial microbial composition was relatively similar throughout. The prevalent phyla were similar to those composing the top 86 cm of the SME borehole, suggesting more aerated conditions may have persisted throughout SHE.

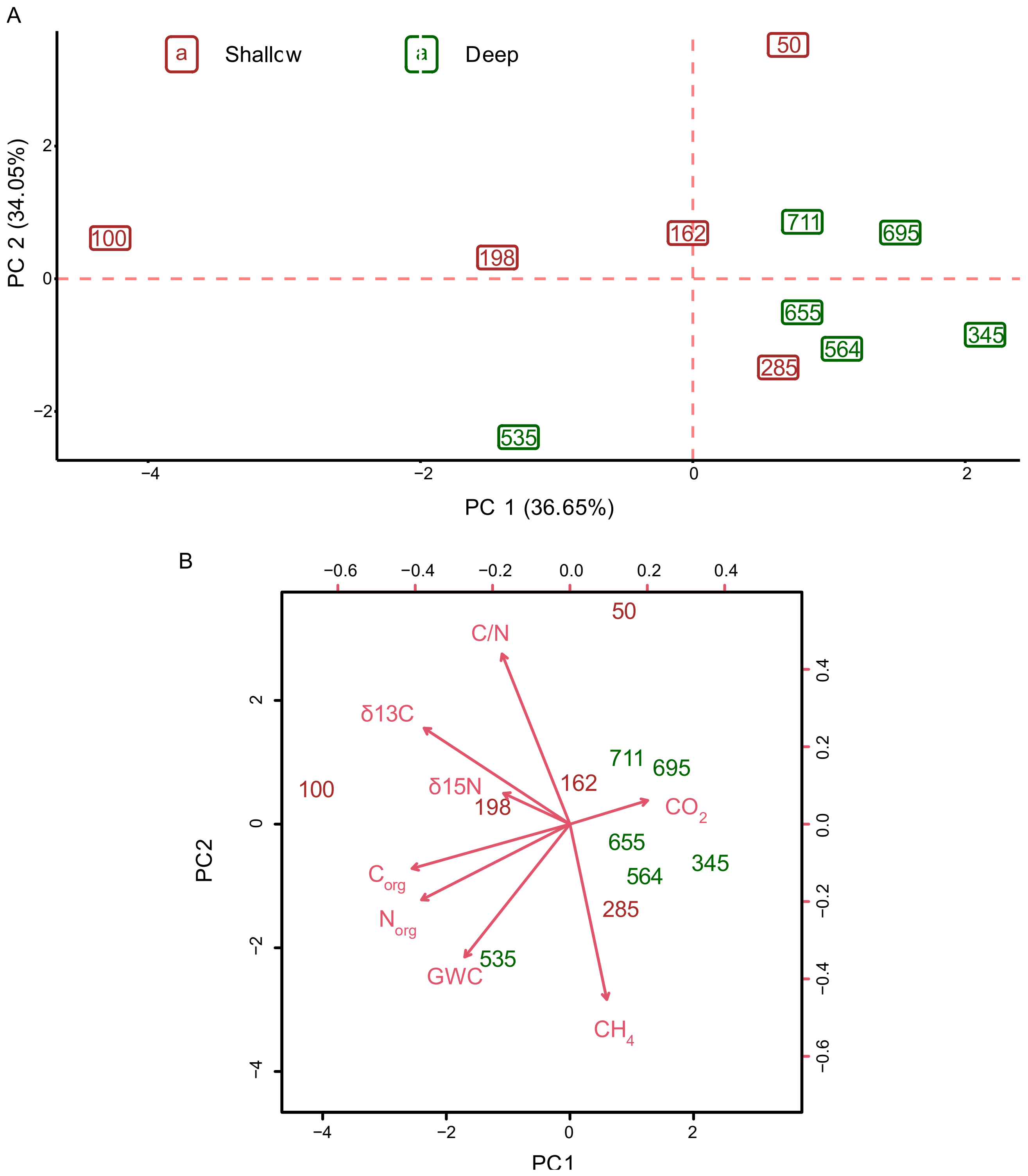

Figure S1: PCA analysis of selected environmental parameters from a full talik profile during winter at the North Star Yedoma study site. (A) PCA analysis (n=11) was performed on shallow (n=5, brown) and deep (n=6, dark green) winter (March 18, 2023) samples (up to ~7 m). **(B)** Loading scores, indicating the importance of tested environmental variables related to PC 1 and PC 2.

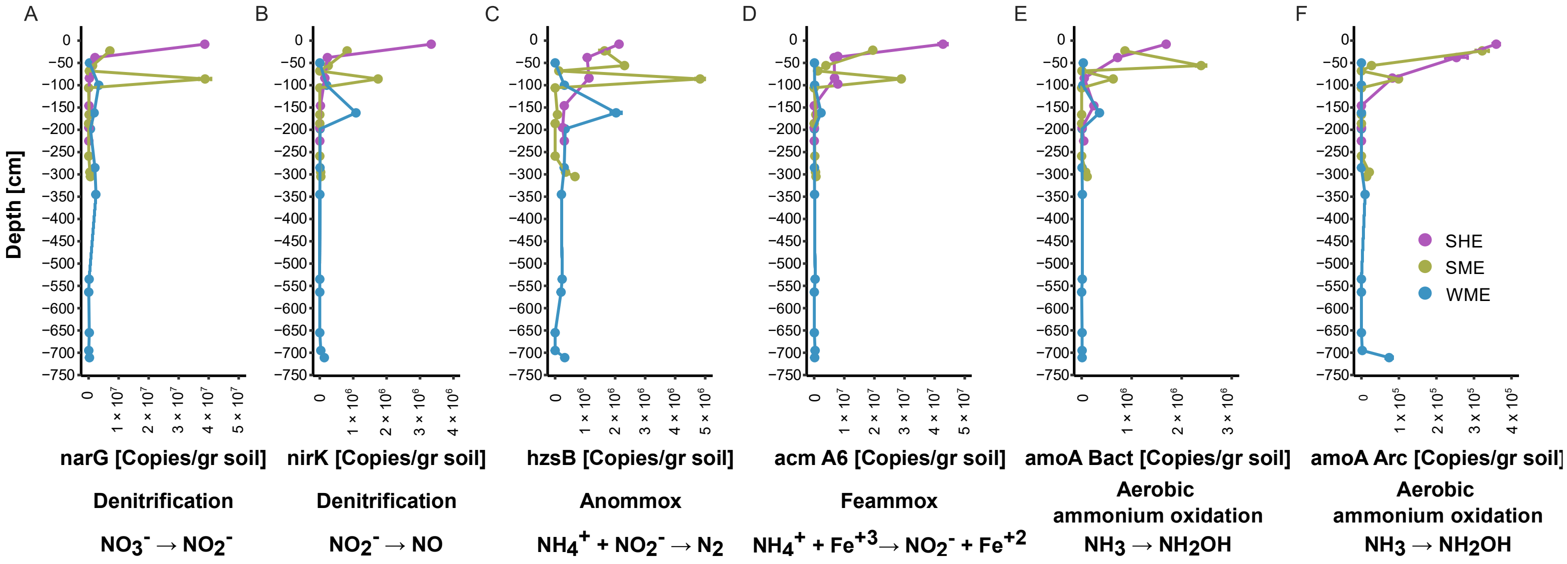

Figure S2. Absolute abundance depth profiles of genes related to the nitrogen cycle. Quantification of absolute abundances (n=15 samples) was performed on shallow summer (September 15, 2021) and winter (March 18, 2023) samples (up to ~3 m), using primers targeting functional genes related to the nitrogen cycle (see Table S11 for a detailed list of genes and primers). The absolute abundance of each gene is presented as copies / gr soil (mean±SE). **(A)** narG (denitrification), **(B)** nirK (denitrification), **(C)** hzsB (Anammox), **(D)** acm A6 (Femmox) **(E)** amoA bacteria and **(F)** amoA archaea (aerobic ammonium oxidation) gene expression. For each gene, the related function and main reaction (main products, unbalanced) are indicated. For the mcrA gene, the double arrow symbol (↔) represents potential forward and reverse methanogenesis. Nitrous oxide and CH_4 concentrations are also presented. SME = Summer Mid Elevation (ID: BH1, n=10, olive-green), WME = Winter Mid Elevation (BH6, n=5, blue).

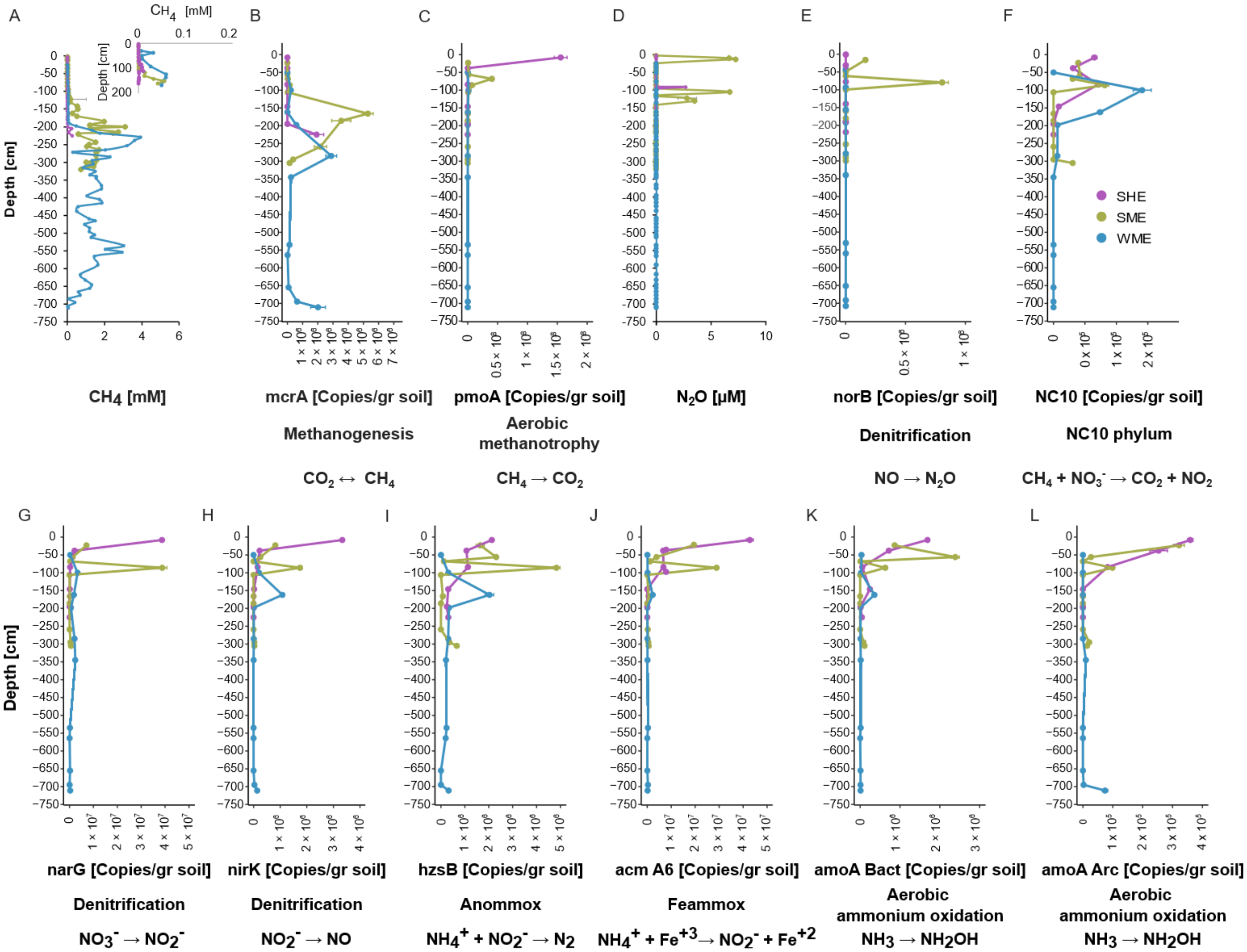

Figure S3. Absolute abundance depth profiles of genes related to the nitrogen and CH_4 cycles. Absolute abundances (n=27 samples) were quantified using qPCR with primers targeting functional genes related to the nitrogen and CH_4 cycles (see Table S11 for a detailed list of genes and primers). The absolute abundance of each gene is presented as copies / gr soil (mean±SE). **(A)** CH4 concentrations in summer and winter boreholes depth profiles. **(B-C)** mcrA and pmoA gene expression **(D)** N2O concentrations in summer and winter boreholes depth profiles. **(E)** norB, **(F)** NC10 phylum, **(G)** narG (denitrification), **(H)** nirK (denitrification), **(I)** hzsB (Anammox), **(J)** acm A6 (Femmox) **(K)** amoA bacteria and **(L)** amoA archaea (aerobic ammonium oxidation) gene expression. For each gene, the related function and main reaction (main products, unbalanced) are indicated. For the mcrA gene, the double arrow symbol (↔) represents potential forward and reverse methanogenesis. nitrous oxide and CH_4 concentrations are also presented. SME = Summer Mid Elevation (borehole ID: BH1, n=10, olive-

green), SHE = Summer High Elevation (BH2, n=6, purple), WME = Winter Mid Elevation (BH6, n=11, blue). Summer sampling refers to cores taken on September 15, 2021 and winter on March 18, 2023.

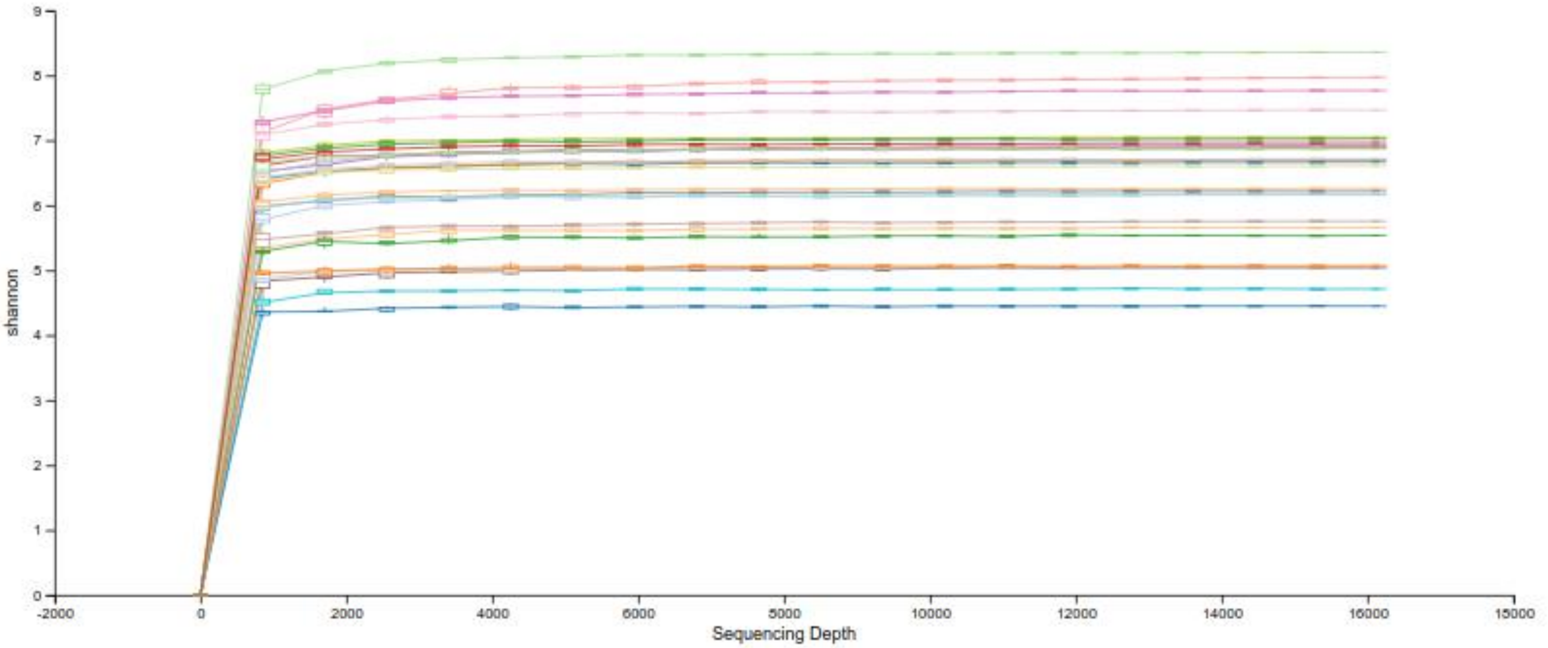

Figure S4. Rarefaction curves for 16S rRNA gene amplicon-based sequencing. Amplicon Sequence Variants (ASVs) were pre-filtered to a minimum frequency of 20 reads across all samples, in a minimum of 2 samples. Rarefaction curves were generated using the q2-alpha-rarefaction function of the q2-diversity plugin. The colors represent the different samples.

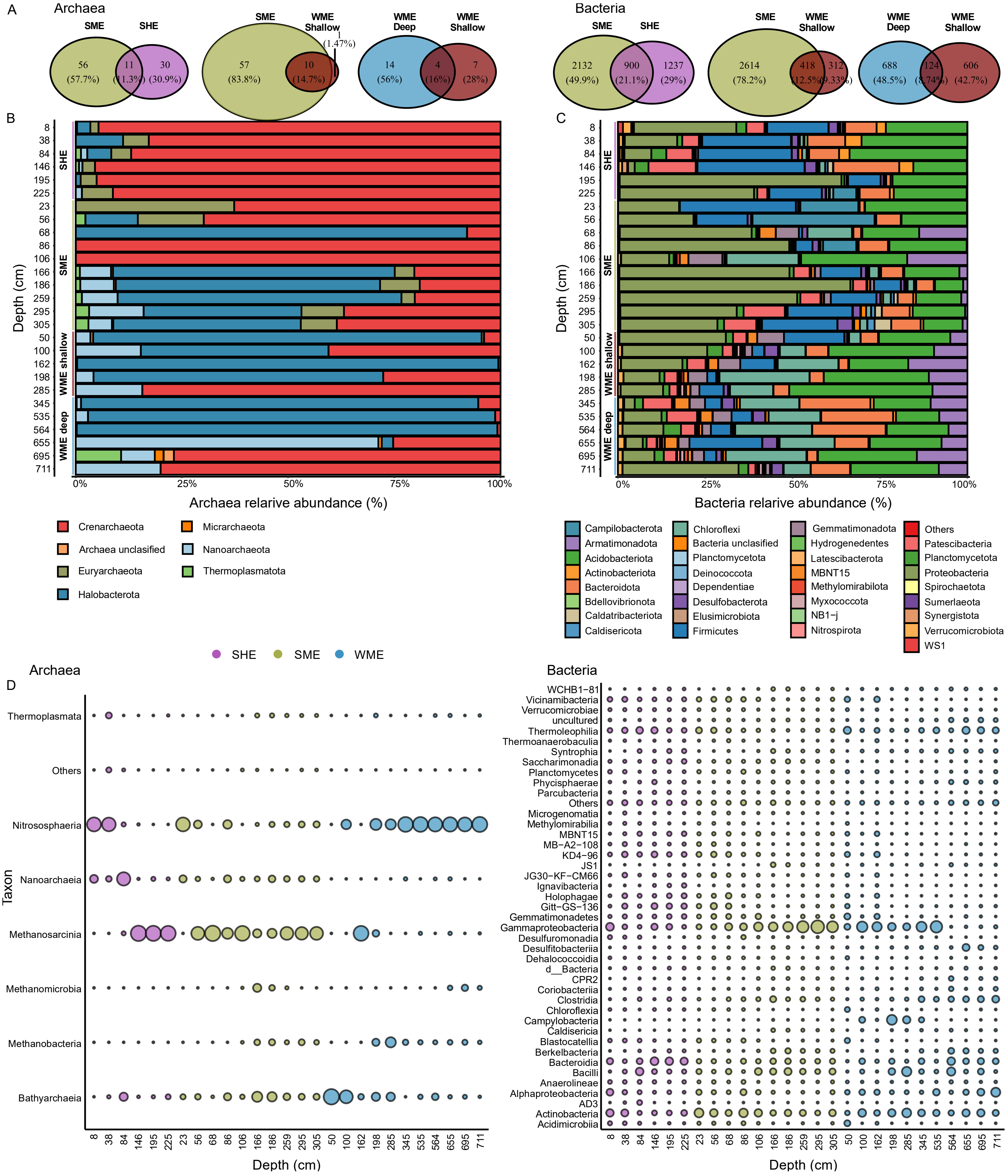

A
Archaea
SME
SHE
56 (57.7%)
11 (11.3%)
30 (30.9%)
SME
WME Shallow
57 (83.8%)
10 (14.7%)
1 (1.47%)
WME Deep
WME Shallow
14 (56%)
4 (16%)
7 (28%)
Bacteria
SME
SHE
2132 (49.9%)
900 (21.1%)
1237 (29%)
SME
WME Shallow
2614 (78.2%)
418 (12.5%)
312 (9.33%)
WME Deep
WME Shallow
688 (48.5%)
124 (8.74%)
606 (42.7%)
B
C
Depth (cm)
SHE
SME
WME shallow
WME deep
Archaea relarive abundance (%)
Bacteria relarive abundance (%)
Crenarchaeota
Micrarchaeota
Archaea unclasified
Nanoarchaeota
Euryarchaeota
Thermoplasmatota
Halobacterota
Campilobacterota
Armatimonadota
Acidobacteriota
Actinobacteriota
Bacteroidota
Bdellovibrionota
Caldatribacteriota
Caldisericota
Chloroflexi
Bacteria unclasified
Planctomycetota
Deinococcota
Dependentiae
Desulfobacterota
Elusimicrobiota
Firmicutes
Gemmatimonadota
Hydrogenedentes
Latescibacterota
MBNT15
Methylomirabilota
Myxococcota
NB1−j
Nitrospirota
Others
Patescibacteria
Planctomycetota
Proteobacteria
Spirochaetota
Sumerlaeota
Synergistota
Verrucomicrobiota
WS1
SHE
SME
WME
D
Archaea
Taxon
Thermoplasmata
Others
Nitrososphaeria
Nanoarchaeia
Methanosarcinia
Methanomicrobia
Methanobacteria
Bathyarchaeia
Depth (cm)
Bacteria
WCHB1−81
Vicinamibacteria
Verrucomicrobiae
uncultured
Thermoleophilia
Thermoanaerobaculia
Syntrophia
Saccharimonadia
Planctomycetes
Phycisphaerae
Parcubacteria
Others
Microgenomatia
Methylomirabilia
MBNT15
MB−A2−108
KD4−96
JS1
JG30−KF−CM66
Ignavibacteria
Holophagae
Gitt−GS−136
Gemmatimonadetes
Gammaproteobacteria
Desulfuromonadia
Desulfitobacteriia
Dehalococcoidia
d__Bacteria
CPR2
Coriobacteriia
Clostridia
Chloroflexia
Campylobacteria
Caldisericia
Blastocatellia
Berkelbacteria
Bacteroidia
Bacilli
Anaerolineae
Alphaproteobacteria
AD3
Actinobacteria
Acidimicrobiia
Depth (cm)

Figure S5. Shifts in microbial community composition at North Star Yedoma. Analysis of community composition was based on 16S rRNA gene amplicon-based sequencing. Three cores were included in the analysis. SME = Summer Mid Elevation (borehole ID: BH1, n=10, olive-green), SHE = Summer High Elevation (BH2, n=6, purple), WME = Winter Mid Elevation (BH6, n=11, blue). When analyzing the full talik, WME samples were further subdivided into shallow (<3 m, n = 5, brown) and deep (>3 m, n = 6, blue) samples. Summer sampling refers to cores taken on September 15, 2021 and winter on March 18, 2023. **(A)** Venn diagram visually representing the number of shared and unique ASVs. Three comparisons were made, between: 1. High and mid elevation (SHE vs. SME, at the top 3 m), 2. Seasonal comparison of Summer vs. winter (SME vs. WME, at the top 3 m), and 3. WME Shallow vs. deep samples of the complete talik. **(B+C)** Relative abundance barplot visualization of archaeal **(B)** and bacterial **(C)** ASVs (minimum frequency ≥1%). Samples are ordered on the Y axis according to borehole and depth. **(D)** Bubble plot representing class level relative abundances of archaea (left panel) and bacteria (right panel). Samples are ordered on the X axis according to borehole and depth. Bubble size indicates relative abundance.

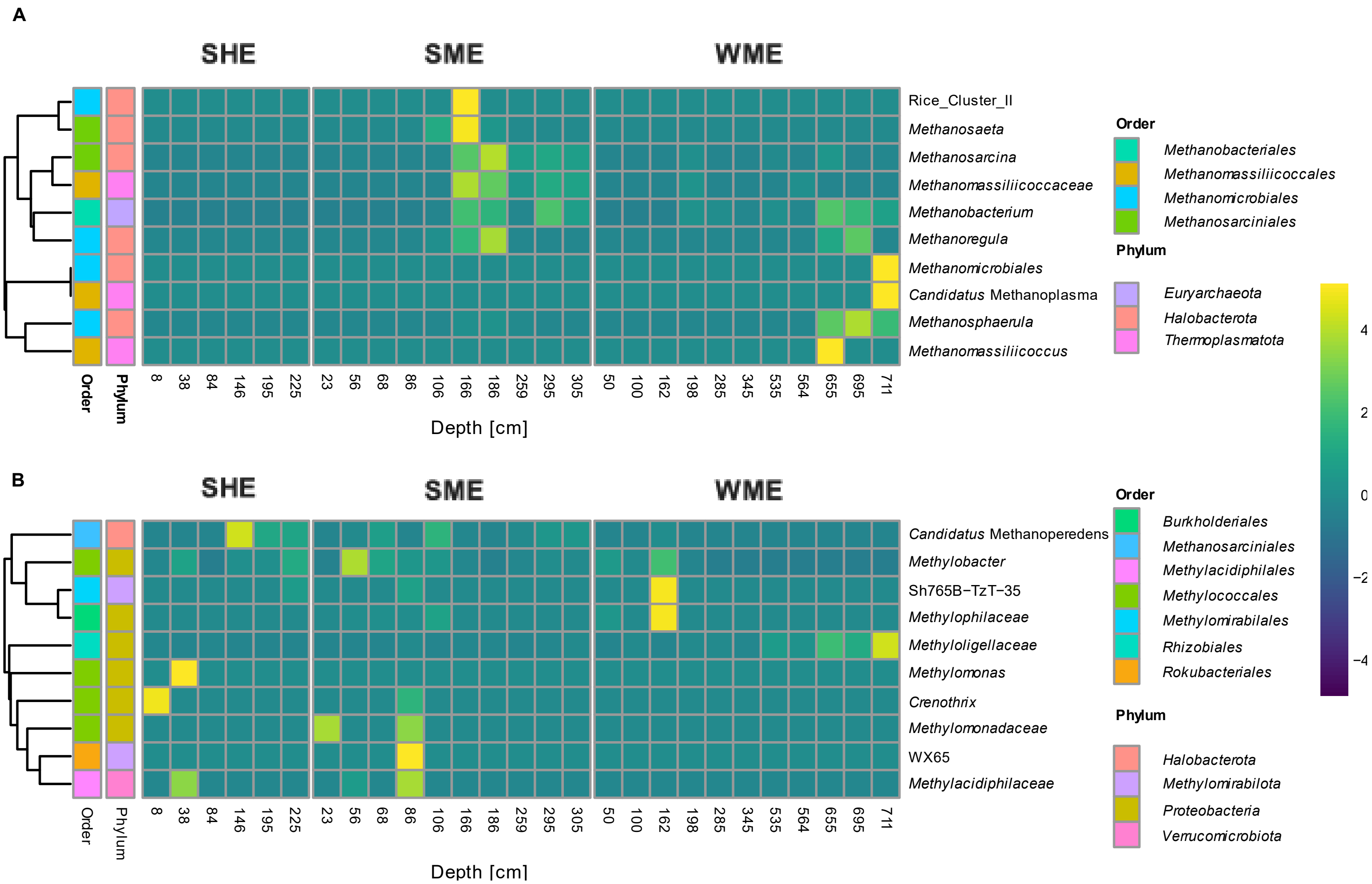

Figure S6. Shifts in the methanogenic and methanotrophic microbial communities at North Star Yedoma. Analysis of microbial community composition was based on 16S rRNA gene amplicon-based sequencing. Heatmap visualizations present relative abundance of methanogenic **(A)** and methanotrophic **(B)** taxa. For each taxon Order and Phylum are presented to the left of the heatmaps. Samples (columns) were ordered according to depth and faceted according to sampling environment. SME = Summer Mid Elevation (borehole ID: BH1, n=10, olive-green), SHE = Summer High Elevation (BH2, n=6, purple), WME = Winter Mid Elevation (BH6, n=11, blue). The Color scale represents relative abundance of ASVs within each sample, ranging from low (blue to black) to high (yellow to green).

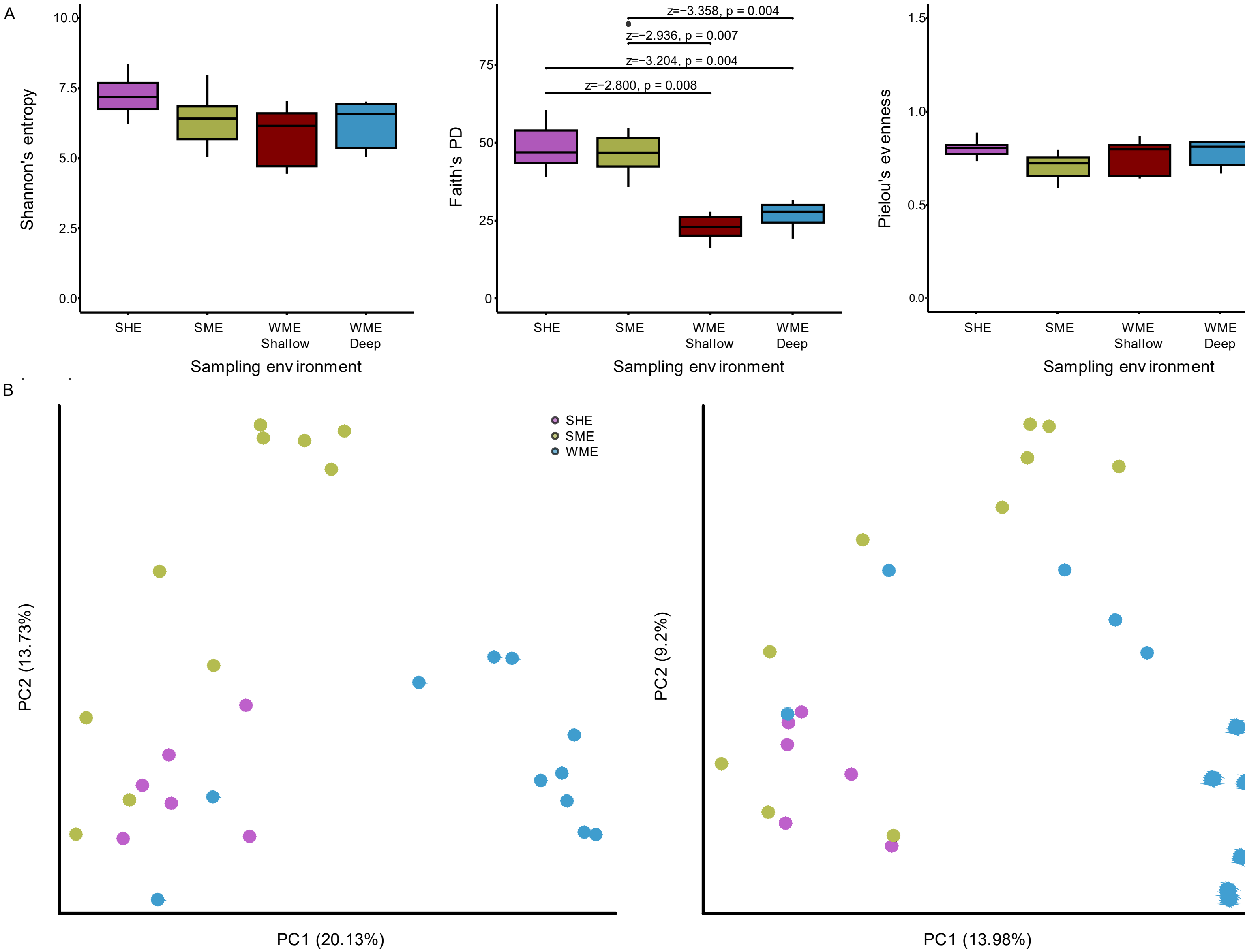

Figure S7. Microbial diversity North Star Yedoma (NSY). Analysis of microbial community composition was based on 16S rRNA gene amplicon-based sequencing. **(A)** Alpha diversity analysis: Shannon's entropy (left panel), Faith's PD (midle panel) and Pielou's evenness (right panel) are presented. Kruskal-Wallis test results and post hoc via Wilcox tests are presented in Supplementary Tables S6-S7. **(B)** Beta diversity was assessed via Jaccard (left panel) and Bray–Curtis (right panel). Statistical significance was tested via PERMANOVA and PERMDISP analyses (Tables S8-S9). SME = Summer Mid Elevation (borehole ID: BH1, n=10, olive-green), SHE = Summer High Elevation (BH2, n=6, purple), WME = Winter Mid Elevation (BH6, n=11). For comparison of alpha diversity full talik WME samples were subdivided to shallow (n=5, brown) and deep (n=6, blue) samples. Summer sampling refers to cores taken on the September 15, 2021 and winter on March 18, 2023.

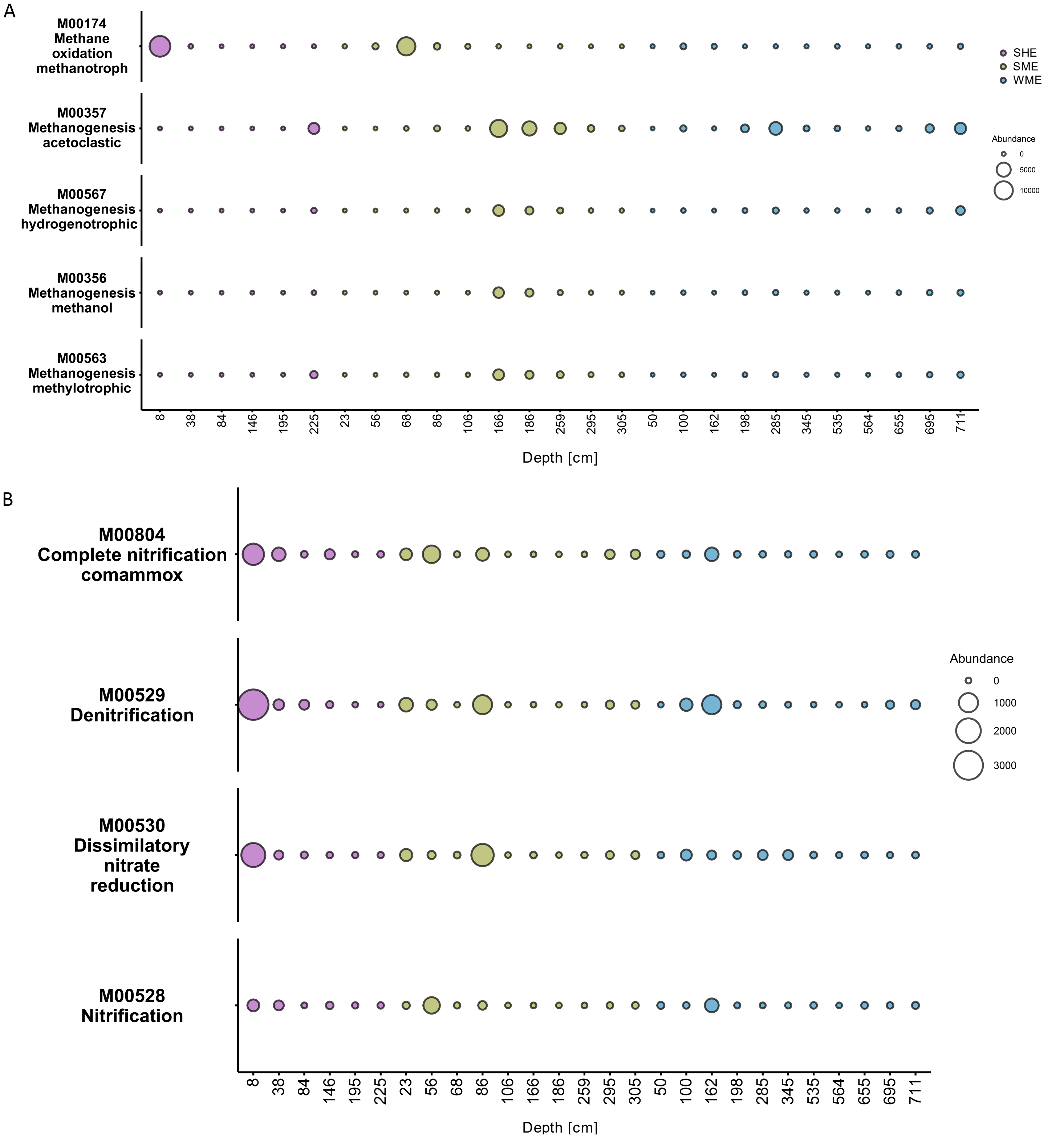

Figure S8. The abundance of CH_4 and nitrogen cycles pathway prediction analysis. Based on 16S rRNA gene amplicon-based sequencing. Amplicon Sequence Variants (ASVs) were pre-filtered to

a minimum frequency of 20 reads across all samples, in a minimum of 2 samples. Raw data was analyzed with the PICRUSt2 package, corrected for qPCR resulats (see material and methods section for a detailed description). **(A)** Methane pathway prediction analysis and **(B)** Nitrogen pathway prediction analysis. To maintain the three main axes of comparison, we have denoted the sampled cores by season and elevation, as follows: SME = Summer Mid Elevation (borehole ID: BH1, n=10, olive-green), SHE = Summer High Elevation (BH2, n=6, purple), WME = Winter Mid Elevation (BH6, n=11, blue). Summer sampling refers to cores taken on the September 15, 2021 and winter on March 18, 2023.

Table S1: Physicochemical characteristics of North Star Yedoma (NSY) samples, sent for microbiological samples. Included variables were: variables volumetric water content (VWC), gravimetric water content (GWC), CH_4 and CO_2 concentrations, C_{org} (%), N_{org} (%), C_{org}/N_{org} ration, $\delta^{15}N$ (‰), $\delta^{13}C$ (‰). In addition, for each sample the following parameters are presented: Sampling depth, season, stratification period. Samples were characterized based on sampling environments: SHE = Summer Shallow High Elevation (n=6), SLE = Summer Shallow Low Elevation (n=10), WLE = Winter Low Elevation (n=11). The WLE samples were comprised of shallow (<3 m, n=5) and deep (<3 m, n=6) samples. For elaboration, see materials and methods.

Sample ID	Borehole	Environment	season	Depth (cm)	VWC (%)	CH_4 (mM)	CO_2 (mM)	GWC (%)	N_{org} (%)	C_{org} (%)	C_{org} / N_{org} (ratio)	$\delta^{15}N$ (‰)	$\delta^{13}C$ (‰)
BH2_8	BH2	SHE	summer	8	31	0	5.2	25	0.16	2.13	13.6	1.7	26.3
BH2_38	BH2	SHE	summer	38	26.3	0	7.2	23	0.22	2.62	12	3.3	26.4
BH2_84	BH2	SHE	summer	84	37.2	0.019	12.1	26	0.16	3.32	20.2	4.5	26.8
BH2_146	BH2	SHE	summer	146	24.8	0.004	5.8	20	0.08	0.7	8.9	0.7	25.3
BH2_195	BH2	SHE	summer	195	26	0.003	0.012	21	0.06	0.55	9.2	4.2	25.3
BH2_225	BH2	SHE	summer	225	28.5	0.061	0.085	23	0.03	0.31	9.6	4.2	24.9
BH1_23	BH1	SLE	summer	23	38.4	0	4.1	25	0.09	1.37	15.5	1.7	26.4
BH1_56	BH1	SLE	summer	56	32.4	0	4.9	23	0.07	1.06	14.2	3.2	26
BH1_68	BH1	SLE	summer	68	32.9	0	5.8	24	0.1	1.32	13	3.1	26.8
BH1_86	BH1	SLE	summer	86	41.5	0	4.8	32	0.11	1.45	13.5	3.1	27
BH1_105	BH1	SLE	summer	106	35.8	0.043	7.6	29	0.17	2.5	14.3	2.6	26.7
BH1_166	BH1	SLE	summer	166	35	0.274	5.2	32	0.31	4.03	12.9	2.7	0
BH1_185	BH1	SLE	summer	186	27.5	1.967	4.8	26	0.09	1.01	11.5	0.4	25.6
BH1_259	BH1	SLE	summer	259	27.2	1.051	1.1	26	0.18	2.1	11.5	2.6	25.6
BH1_295	BH1	SLE	summer	295	27.6	1.572	3	27	0.21	2.33	11	1.8	25.9
BH1_305	BH1	SLE	summer	305	28.9	1.439	2.6	28	0.07	0.95	12.6	0.5	26
BH6_50	BH6	WLE	winter	50	22	0.008	0.8	15	0.03	0.51	15.06	2.98	26.17
BH6_100	BH6	WLE	winter	100	28	0.059	0	27	0.19	2.45	12.89	4.5	27.08
BH6_162	BH6	WLE	winter	162	30	0.047	7.7	24	0.09	1.05	11.61	2.98	25.68
BH6_198	BH6	WLE	winter	198	25	0.474	0	28	0.09	1.1	12.5	4.25	25.99

BH6_285	BH6	WLE	winter	285	28	2.279	0	25	0.06	0.57	9.88	7.24	25.06
BH6_345	BH6	WLE	winter	345	29	1.539	2.7	21	0.06	0.63	10.48	1.28	24.66
BH6_535	BH6	WLE	winter	535	30	3.017	0	27	0.16	1.63	10.46	1.9	25.46
BH6_564	BH6	WLE	winter	564	31	1.465	3.9	24	0.08	0.86	10.31	1.75	25.13
BH6_655	BH6	WLE	winter	655	26	1.194	0	21	0.07	0.76	10.62	3.44	24.98
BH6_695	BH6	WLE	winter	695	25	0.4	3.9	18	0.06	0.66	10.73	2.95	25.25
BH6_711	BH6	WLE	winter	711	26	0.044	0	18	0.07	0.77	11.13	2.82	25.07

Table S2: Correlations between the physicochemical parameters comprising the PCA analyses. Included variables were: Gravimetric Water Content (GWC), CH_4, CO_2, C_{org}, N_{org}, C_{org}/N_{org} ration, $\delta^{15}N$, $\delta^{13}C$.

Comparison	Variable 1	Variable 2	Corr	p-value
Season	N_{org}	C_{org}	0.934	0.000
Season	CH_4	C/N	-0.864	0.000
Season	CH_4	$\delta^{13}C$	-0.687	0.005
Season	C/N	$\delta^{13}C$	0.648	0.009
Season	GWC	C_{org}	0.605	0.017
Season	GWC	N_{org}	0.597	0.019
Season	CH_4	CO_2	-0.443	0.099
Season	CO_2	C/N	0.303	0.273
Season	CO_2	$\delta^{15}N$	-0.299	0.278
Season	CO_2	C_{org}	0.255	0.360
Season	CH_4	$\delta^{15}N$	-0.235	0.399
Season	CH_4	GWC	0.233	0.404
Season	CO_2	N_{org}	0.224	0.423
Season	$\delta^{15}N$	$\delta^{13}C$	0.211	0.451
Season	C_{org}	$\delta^{13}C$	0.179	0.524
Season	GWC	$\delta^{15}N$	-0.175	0.534
Season	CH_4	C_{org}	-0.164	0.559
Season	N_{org}	$\delta^{15}N$	-0.164	0.560
Season	C_{org}	C/N	0.138	0.625
Season	N_{org}	C/N	-0.122	0.665
Season	GWC	C/N	-0.092	0.745
Season	C_{org}	$\delta^{15}N$	-0.073	0.795
Season	N_{org}	$\delta^{13}C$	0.039	0.891
Season	CO_2	$\delta^{13}C$	0.037	0.896
Season	CH_4	N_{org}	0.036	0.898
Season	C/N	$\delta^{15}N$	0.030	0.917
Season	GWC	$\delta^{13}C$	0.019	0.947
Season	CO_2	GWC	-0.004	0.990
Full talik	N_{org}	C_{org}	0.986	0.000

Full talik	CH_4	C/N	-0.845	0.001
Full talik	GWC	N_{org}	0.765	0.006
Full talik	C/N	$\delta^{13}C$	0.736	0.010
Full talik	GWC	C_{org}	0.716	0.013
Full talik	CH_4	$\delta^{13}C$	-0.509	0.110
Full talik	CH_4	GWC	0.500	0.117
Full talik	C_{org}	$\delta^{13}C$	0.473	0.142
Full talik	CO_2	$\delta^{15}N$	-0.468	0.146
Full talik	N_{org}	$\delta^{13}C$	0.438	0.178
Full talik	CO_2	GWC	-0.386	0.241
Full talik	GWC	$\delta^{15}N$	0.361	0.275
Full talik	$\delta^{15}N$	$\delta^{13}C$	0.355	0.284
Full talik	C/N	$\delta^{15}N$	0.314	0.346
Full talik	GWC	$\delta^{13}C$	0.284	0.397
Full talik	CO_2	N_{org}	-0.244	0.469
Full talik	CO_2	C_{org}	-0.219	0.518
Full talik	CH_4	CO_2	-0.204	0.548
Full talik	CH_4	$\delta^{15}N$	-0.173	0.611
Full talik	C_{org}	C/N	0.173	0.612
Full talik	GWC	C/N	-0.151	0.657
Full talik	N_{org}	$\delta^{15}N$	0.122	0.720
Full talik	CH_4	N_{org}	0.120	0.726
Full talik	N_{org}	C/N	0.115	0.736
Full talik	CH_4	C_{org}	0.064	0.853
Full talik	C_{org}	$\delta^{15}N$	0.059	0.863
Full talik	CO_2	C/N	-0.005	0.988
Full talik	CO_2	$\delta^{13}C$	0.005	0.988
Elevation	N_{org}	C_{org}	0.924	0.000
Elevation	C/N	$\delta^{13}C$	0.722	0.002
Elevation	CO_2	C_{org}	0.591	0.016
Elevation	GWC	C_{org}	0.548	0.028
Elevation	C_{org}	C/N	0.528	0.035

Elevation	CH_4	$\delta^{13}C$	-0.526	0.036
Elevation	GWC	N_{org}	0.521	0.038
Elevation	CO_2	C/N	0.511	0.043
Elevation	CO_2	$\delta^{13}C$	0.510	0.044
Elevation	CO_2	N_{org}	0.462	0.071
Elevation	CH_4	C/N	-0.422	0.104
Elevation	CH_4	GWC	0.407	0.118
Elevation	CH_4	$\delta^{15}N$	-0.398	0.127
Elevation	GWC	C/N	0.390	0.135
Elevation	C_{org}	$\delta^{13}C$	0.372	0.156
Elevation	CH_4	CO_2	-0.329	0.213
Elevation	GWC	$\delta^{15}N$	-0.267	0.318
Elevation	GWC	$\delta^{13}C$	0.255	0.340
Elevation	N_{org}	C/N	0.237	0.377
Elevation	N_{org}	$\delta^{13}C$	0.212	0.430
Elevation	$\delta^{15}N$	$\delta^{13}C$	0.155	0.568
Elevation	C/N	$\delta^{15}N$	0.147	0.588
Elevation	C_{org}	$\delta^{15}N$	0.118	0.664
Elevation	CH_4	N_{org}	0.095	0.728
Elevation	CO_2	$\delta^{15}N$	0.086	0.752
Elevation	CO_2	GWC	0.069	0.799
Elevation	N_{org}	$\delta^{15}N$	-0.053	0.845
Elevation	CH_4	C_{org}	-0.035	0.898

Table S3: Loading scores of environmental variables included in the season (Fig. 2) PCA analysis. Included variables were: Gravimetric Water Content (GWC), CH_4, CO_2, C_{org}, N_{org}, C_{org}/N_{org} ration, $\delta^{15}N$, $\delta^{13}C$.

Parameter	PC1	PC2
CH_4	0.037	0.650
CO_2	-0.206	-0.330
GWC	-0.421	0.198
N_{org}	-0.543	0.067
C_{org}	-0.543	-0.056
C/N	0.023	-0.648
$\delta^{15}N$	0.068	-0.039
$\delta^{13}C$	0.429	0.034

Table S4: Loading scores of environmental variables included in the full talik profile based PCA analyses. Included variables were: volumetric gravimetric water content (GWC), CH_4, CO_2, C_{org}.

Parameter	PC1	PC2
CH_4	0.119	-0.567
CO_2	0.251	0.076
GWC	-0.340	-0.429
N_{org}	-0.480	-0.245
C_{org}	-0.510	-0.143
C/N	-0.219	0.550
$\delta^{15}N$	-0.215	0.100
$\delta^{13}C$	-0.471	0.310

Table S5: peaks of qPCR values related to the CH_4 and nitrogen cycles, in the four sampled environments. For each environment, the peak expression is presented along with the corresponding depth. Samples were characterized based on sampling environments: SHE = Summer Shallow High Elevation (n=6), SLE = Summer Shallow Low Elevation (n=10), WLE = Winter Low Elevation (n=11). The WLE samples were comprised of shallow (<3 m, n=5) and deep (<3 m, n=6) samples. For elaboration, see materials and methods. p-value considered significant if < 0.05, and marked bold with underline.

Gene	Process	SHE peak	SLE peak	WLE peak Shallow samples (<3 m)	WLE peak Deep samples (>3 m)
		Expression (depth) (copies/1gr soil)	Expression (depth) (copies/1gr soil)	Expression (depth) (copies/1gr soil)	Expression (depth) (copies/1gr soil)
**mcrA**	**Methanogenesis**	$1.9x10^{6} \pm 4.7x10^{5}$ (at 225 cm)	$5.3x10^{6} \pm 3.5x10^{5}$ (at 166 cm)	$2.8x10^{6} \pm 3.6x10^{5}$ (at 285 cm)	$2x10^{6} \pm 4.9x10^{5}$ (at 711 cm)
**pmoA**	**Aerobic CH_4 oxidation**	$15.6x10^{7} \pm 10.6x10^{6}$ (at 8 cm)	$4.05x10^{7} \pm 1.02x10^{6}$ (at 68 cm)	$1.4\ x10^{6} \pm 3.3x10^{5}$ (at 100 cm)	$1.4x10^{5} \pm 1.8x10^{4}$ (at 711 cm)
**narG**	**Denitrification**	$3.9x10^{7} \pm 7.5x10^{5}$ (at 8 cm)	$3.9x10^{7} \pm 2x10^{6}$ (at 86 cm)	$3.4x10^{6} \pm 1.6x10^{5}$ (at 100 cm)	$2.5x10^{6} \pm 6.6x10^{4}$ (at 345 cm)
**nirK**	**Denitrification**	$3.3x10^{6} \pm 4.5x10^{4}$ (at 8 cm)	$1.7x10^{6} \pm 8.6x10^{4}$ (at 86 cm)	$1.1x10^{6} \pm 5.2x10^{4}$ (at 162 cm)	$1.4x10^{5} \pm 2x10^{4}$ (at 711 cm)
**norB**	**Denitrification**	0	$2.3\ x10^{5} \pm 1.5x10^{4}$ (at 86 cm depth)	0	0
**hzsB**	**Anammox**	$2.1x10^{6} \pm 6.8x10^{4}$ (at 8 cm)	$6.8x10^{6} \pm 1.5x10^{5}$ (at 86 cm)	$2x10^{6} \pm 1.8x10^{3}$ (at 162 cm)	$3.2x10^{5} \pm 3x10^{4}$ (at 711 cm)
**A6-acm (16S)**	**Feammox**	$4.3x10^{7} \pm 1.5x10^{6}$ (at 8 cm)	$2.9x10^{7} \pm 6.8x10^{5}$ (at 86 cm)	$2.3x10^{6} \pm 1.4x10^{5}$ (at 162 cm)	$3.2x10^{5} \pm 2x10^{4}$ (at 535 cm)
**NC10 (16S)**	**NC10 phylum**	$6.5x10^{4} \pm 4.2x10^{3}$ (at 8 cm)	$8.1x10^{4} \pm 6.4x10^{3}$ (at 86 cm)	$1.4x10^{5} \pm 1.4x10^{4}$ (at 100 cm)	0
**amoA archaea**	**Aerobic ammonium oxidation**	$3.6x10^{5} \pm 9.4x10^{3}$ (at 8 cm)	$3.2x10^{5} \pm 1.7x10^{4}$ (at 23 cm)	0	$7.3x10^{4} \pm 9.2x10^{3}$ (at 711 cm)
**amoA bacteria**		$1.7x10^{6} \pm 3.8x10^{4}$ (at 8 cm)	$2.4x10^{6} \pm 1x10^{5}$ (at 56 cm)	$3.6x10^{5} \pm 1.9x10^{4}$ (at 162 cm)	$1.4x10^{4} \pm 1.7x10^{3}$ (at 345 cm)

Table S6: Kruskal-Wallis rank sum tests for 16S NGS alpha diversity between sampled environments: SHE = Summer Shallow High Elevation (n=6), SLE = Summer Shallow Low Elevation (n=10), WLE = Winter Low Elevation (n=11). The WLE samples were comprised of shallow (<3 m, n=5) and deep (<3 m, n=6) samples. For elaboration, see materials and methods. p-value considered significant if < 0.05, and marked bold with underline.

Comparison	Alpha index	df	W statistic	p-value
**Sampling environment**	**Shannon's entropy**	3	5.41	0.144
	**Faith's PD**	3	19.18	**0.0002**
	**Pielou's evenness**	3	6.9	**0.074**

Table S7: Post hoc via Dunn's tests of 16S NGS alpha diversity between sampled environments: SHE = Summer Shallow High Elevation (n=6), SLE = Summer Shallow Low Elevation (n=10), WLE = Winter Low Elevation (n=11). The WLE samples were comprised of shallow (WSLE, <3 m, n=5) and deep (WDLE, <3 m, n=6) samples. p-value considered significant if < 0.05, and marked bold with underline.

Alpha index	group1	group2	n1	n2	statistic	p-value	p.adj
Faith pd	SHE	SLE	6	10	-0.195	0.845	0.845
	SHE	WDLE	6	5	-2.800	0.005	**<u>0.008</u>**
	SHE	WSLE	6	6	-3.204	0.001	**<u>0.004</u>**
	SLE	WDLE	10	5	-2.936	0.003	**<u>0.007</u>**
	SLE	WSLE	10	6	-3.358	0.001	**<u>0.004</u>**
	WDLE	WSLE	5	6	-0.534	0.593	0.712

Table S8: PERMANOVA and PERMDISP results of 16S NGS beta diversity. Samples were grouped based on sampling environments: SHE = Summer Shallow High Elevation (n=6), SLE = Summer Shallow Low Elevation (n=10), WLE = Winter Low Elevation (n=11). The WLE samples were comprised of shallow (WSLE, <3 m, n=5) and deep (WDLE, <3 m, n=6) samples. For elaboration, see materials and methods. Number of permutations = 999. p-value considered significant if < 0.05, and marked bold with underline.

Beta index	Test	Comparison	n	N (groups)	test statistic	p-value
Jaccard	PERMDISP	Sampling environment	27	4	1.73	0.264
Bray Curtis	PERMDISP		27	4	1.188	0.428
Jaccard	PERMANOVA		27	4	2.402	**0.001**
Bray Curtis	PERMANOVA		27	4	3.798	**0.001**

Table S9: PERMANOVA and PERMDISP post hoc pairwise comparison results of 16S NGS beta diversity. Samples were grouped based on sampling environments: SHE = Summer Shallow High Elevation (n=6), SLE = Summer Shallow Low Elevation (n=10), WLE = Winter Low Elevation (n=11). The WLE samples were comprised of shallow (WSLE, <3 m, n=5) and deep (WDLE, <3 m, n=6) samples. For elaboration, see materials and methods. Number of permutations = 999. p-value considered significant if < 0.05, and marked bold with underline.

Bata index	Test	Comparison	Group 1	Group 2	n	pseudo-F	p-value	q-value
**Jaccard**	**PERMANOVA**	**Sampling environment**	SHE	SLE	16	999	1.787432178	**<u>0.018</u>**
			SHE	WDLE	11	999	3.311730722	**<u>0.003</u>**
			SHE	WSLE	12	999	1.98687807	**<u>0.003</u>**
			SLE	WDLE	15	999	3.271207712	**<u>0.001</u>**
			SLE	WSLE	16	999	1.741631202	**<u>0.008</u>**
			WDLE	WSLE	11	999	2.568510894	**<u>0.001</u>**
**Bray Curtis**	**PERMANOVA**	**Sampling environment**	SHE	SLE	16	999	2.658819688	**<u>0.002</u>**
			SHE	WDLE	11	999	4.501196181	**<u>0.002</u>**
			SHE	WSLE	12	999	2.539094434	**<u>0.007</u>**
			SLE	WDLE	15	999	6.366077494	**<u>0.001</u>**
			SLE	WSLE	16	999	3.422828237	**<u>0.001</u>**
			WDLE	WSLE	11	999	3.221380856	**<u>0.002</u>**

Table S10: ADONIS test results of 16S NGS beta diversity with selected environmental variables. Number of permutations = 999. MGC = Gravimetric Water Content. Variables were chosen based on the principal component analysis (Fig. 3A-B and Table S4). p-value considered significant if < 0.05, and marked bold with underline.

Beta index	Variable	df	SumsOfSqs	MeanSqs	F.Model	R2	Pr(>F)
**Jaccard**	**GWC**	1	0.652	0.652	1.951	0.060	**0.003**
	**ch4**	1	0.910	0.910	2.724	0.084	**0.001**
	**co2**	1	0.445	0.445	1.331	0.041	0.060
	**Corg**	1	0.364	0.364	1.089	0.034	0.273
	**env**	3	2.104	0.701	2.099	0.194	**0.001**
	**Residuals**	19	6.349	0.334	NA	0.587	NA
	**Total**	26	10.823	NA	NA	1.000	NA
**Bray Curtis**	GWC	1	0.839	0.839	3.398	0.088	**0.001**
	ch4	1	0.979	0.979	3.964	0.102	**0.001**
	co2	1	0.364	0.364	1.476	0.038	0.091
	Corg	1	0.281	0.281	1.138	0.029	0.291
	env	3	2.422	0.807	3.271	0.253	**0.001**
	Residuals	19	4.690	0.247	NA	0.490	NA
	Total	26	9.575	NA	NA	1.000	NA

Table S11: Primers and gBlocks used in this study. gBlocks were constructed based on the sequences of relevant gene, denoted by their NCBI accession number. The full references are presented at the main manuscript.

Gene	Biogeochemical Function	Primer name	Sequence (5'-3')	Length (bp)	Primer concentration (mM)	* gBlocks NCBI accession number	References
mcrA	methanogenesis	ME1F<br>ME3R	GCMATGCARATHGGWATGTC<br>TGTGTGAASCCKACDCCACC	350	0.5	NC_014507.1	[107, 108]
pmoA	CH_4 oxidation	A189gc<br>mb661	GGNGACTGGGACTTCTGG<br>CCGGMGCAACGTCYTTACC	472	0.5	L40804.2	[109–111]
nirK	denitrification	F1aCu<br>R3Cu	ATCATGGTSCTGCCGCG<br>GCCTCGATCAGRTTGTGGTT	473	0.15	EF623493.1	[112]
narG	denitrification	narG571F<br>narG773R	CCGATYCCGGCVATGTCSAT<br>GGNACGTTNGADCCCCA	203	0.5	Based on plasmid	[113]
norB	denitrification	qnorB2<br>qnorB5R	GGNCAYCARGGNTAYGA<br>ACCCANAGRTGNACNACCCACCA	263	0.5	Based on plasmid	[114]
amoA bacteria	Aerobic ammonium oxidation	amoA-1F<br>amoA-2R	GGGGTTTCTACTGGTGGT<br>CCCCTCKGSAAAGCCTTCTTC	429	0.5	U76552.1	[115, 116]
amoA archaea	Aerobic ammonium oxidation	Arch-amoA-F<br>Arch-amoA-R	STAATGGTCTGGCTTAGACG<br>GCGGCCATCCATCTGTATGT	635	0.25	MF176967.1	[117, 118]
hzsB	ANAMMOX	HSBeta396F<br>HSBeta742R	ARGGHTGGGGHAGYTGGAAG<br>GTYCCHACRTCATGVGTCTG	385	0.5	KP002830.1	[119, 120]
16S	Feammox (Acidimicrobiaceae sp. strain A6)	acm342f<br>acm439r	GCAATGGGGGAAACCCTGAC<br>ACCGTCAATTTCGTCCCTGC	200	0.15	MG589453.1	[121]
16S	NC10 phylum	qp1F<br>qp1R	GGGCTTGACATCCCACGAACCTG<br>CGCCTTCCTCCAGCTTGACGC	203	0.15	MZ778768.1	[122]
